# Landscape effects on the contemporary genetic structure of Ruffed Grouse (*Bonasa umbellus*) populations

**DOI:** 10.1002/ece3.5112

**Published:** 2019-05-01

**Authors:** Ashley M. Jensen, Nicholas P. O'Neil, Andrew N. Iwaniuk, Theresa M. Burg

**Affiliations:** ^1^ Department of Biological Sciences University of Lethbridge Lethbridge Alberta Canada; ^2^ Canadian Centre for Behavioural Neuroscience University of Lethbridge Lethbridge Alberta Canada

**Keywords:** dispersal barriers, gene flow, isolation by resistance, landscape genetics, population genetics, ruffed grouse

## Abstract

The amount of dispersal that occurs among populations can be limited by landscape heterogeneity, which is often due to both natural processes and anthropogenic activity leading to habitat loss or fragmentation. Understanding how populations are structured and mapping existing dispersal corridors among populations is imperative to both determining contemporary forces mediating population connectivity, and informing proper management of species with fragmented populations. Furthermore, the contemporary processes mediating gene flow across heterogeneous landscapes on a large scale are understudied, particularly with respect to widespread species. This study focuses on a widespread game bird, the Ruffed Grouse (*Bonasa umbellus*), for which we analyzed samples from the western extent of the range. Using three types of genetic markers, we uncovered multiple factors acting in concert that are responsible for mediating contemporary population connectivity in this species. Multiple genetically distinct groups were detected; microsatellite markers revealed six groups, and a mitochondrial marker revealed four. Many populations of Ruffed Grouse are genetically isolated, likely by macrogeographic barriers. Furthermore, the addition of landscape genetic methods not only corroborated genetic structure results, but also uncovered compelling evidence that dispersal resistance created by areas of unsuitable habitat is the most important factor mediating population connectivity among the sampled populations. This research has important implications for both our study species and other inhabitants of the early successional forest habitat preferred by Ruffed Grouse. Moreover, it adds to a growing body of evidence that isolation by resistance is more prevalent in shaping population structure of widespread species than previously thought.

## 1 INTRODUCTION

Dispersal of organisms across the landscape ultimately determines gene flow among populations, and therefore population connectivity (Slatkin, 1985). Gene flow is essential in species perseverance as it maintains the genetic diversity necessary for populations to respond to changing ecological conditions (Frankham, 2005; Reed & Frankham, 2003). Using genetics, one can detect extrinsic factors restricting dispersal, such as mountain ranges (Funk et al., 2005; Worley et al., 2004), bodies of water (Díaz‐Muñoz, 2012), or anthropogenic disturbance (Cegelski, Waits, & Anderson, 2003; Epps et al., 2005). For many species, the structure of the landscape is an important factor shaping contemporary distributions. Unsuitable habitat is a potential barrier to gene flow, but it is not necessarily an impermeable barrier. Habitat often varies in its degree of suitability (Cushman, McKelvey, Hayden, & Schwartz, 2006), resulting in a complex matrix of habitat types with varying dispersal costs or resistance to individuals moving across the landscape. With current landscape genetic methods, it is possible to identify areas of the landscape that are impeding or facilitating connectivity, and also identify the environmental factors that underlie patterns of contemporary gene flow (Keyghobadi, Roland, & Strobek, 1999; Manel, Schwartz, Luikart, & Taberlet, 2003; Storfer et al., 2007).

Differences in landscape resistance and physical distance can both dictate patterns of gene flow (Ruiz‐Gonzalez, Cushman, Madeira, Randi, & Gómez‐Moliner, 2015). When landscape heterogeneity exists between populations, suitable dispersal routes become more complex. For example, if one of two possible dispersal routes requires movement through habitat that is unsuitable for the study species, then it is likely to present more resistance to dispersing individuals than a route through suitable habitat even when geographic distances are the same. For this reason, landscape heterogeneity within a species' range means patterns of isolation by resistance (IBR) are more likely to occur (Fontaine et al., 2007; McRae & Beier, 2007; Ruiz‐Gonzalez et al., 2015). Physical distance between populations can also act as a barrier by creating clinal genetic variation (Ruiz‐Gonzalez et al., 2015), or isolation by distance (IBD), as species dispersal is as a function of geographic distance. IBD and IBR are not, however, mutually exclusive and sometimes a combination of the two best explains genetic structuring (Metzger, Espindola, Waits, & Sullivan, 2015; Piertney, MacColl, Bacon, & Dallas, 1998). Species that are widespread and relatively continuously distributed are expected to exhibit either panmixia or clinal patterns of genetic structure explained by IBD, particularly when comparing populations at a large scale (Alcaide et al., 2009; Purdue, Smith, & Patton, 2000; Ralston & Kirchman, 2012). A few studies have emerged where widespread, continuously distributed species exhibit unexpected patterns of IBR (Pease et al., 2009; Pilot et al., 2006), but the extent to which species with broad geographic ranges exhibit IBD or IBR is unclear (Basto et al., 2016; Frankham, Ballou, & Briscoe, 2010). Understanding the roles that distance and resistance play in structuring populations is dependent on studying both species with limited distributions and broadly ranging species that are not experiencing obvious breaks in population connectivity.

**Figure 1:**
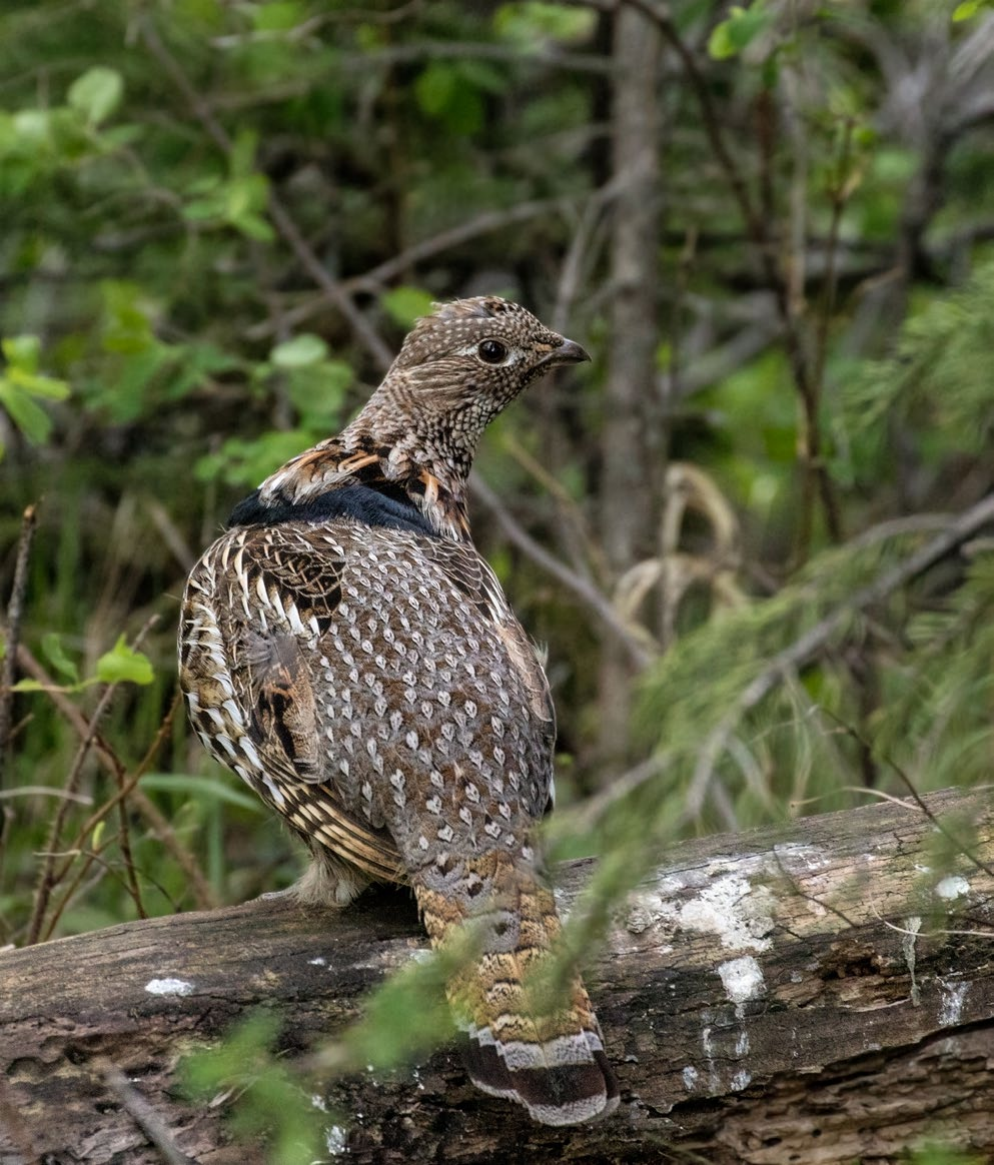
A Ruffed Grouse in the mixed aspen forest of Crowsnest Pass, Alberta, Canada. Copyright:Ashley Jensen (2016)

**Figure 2:**
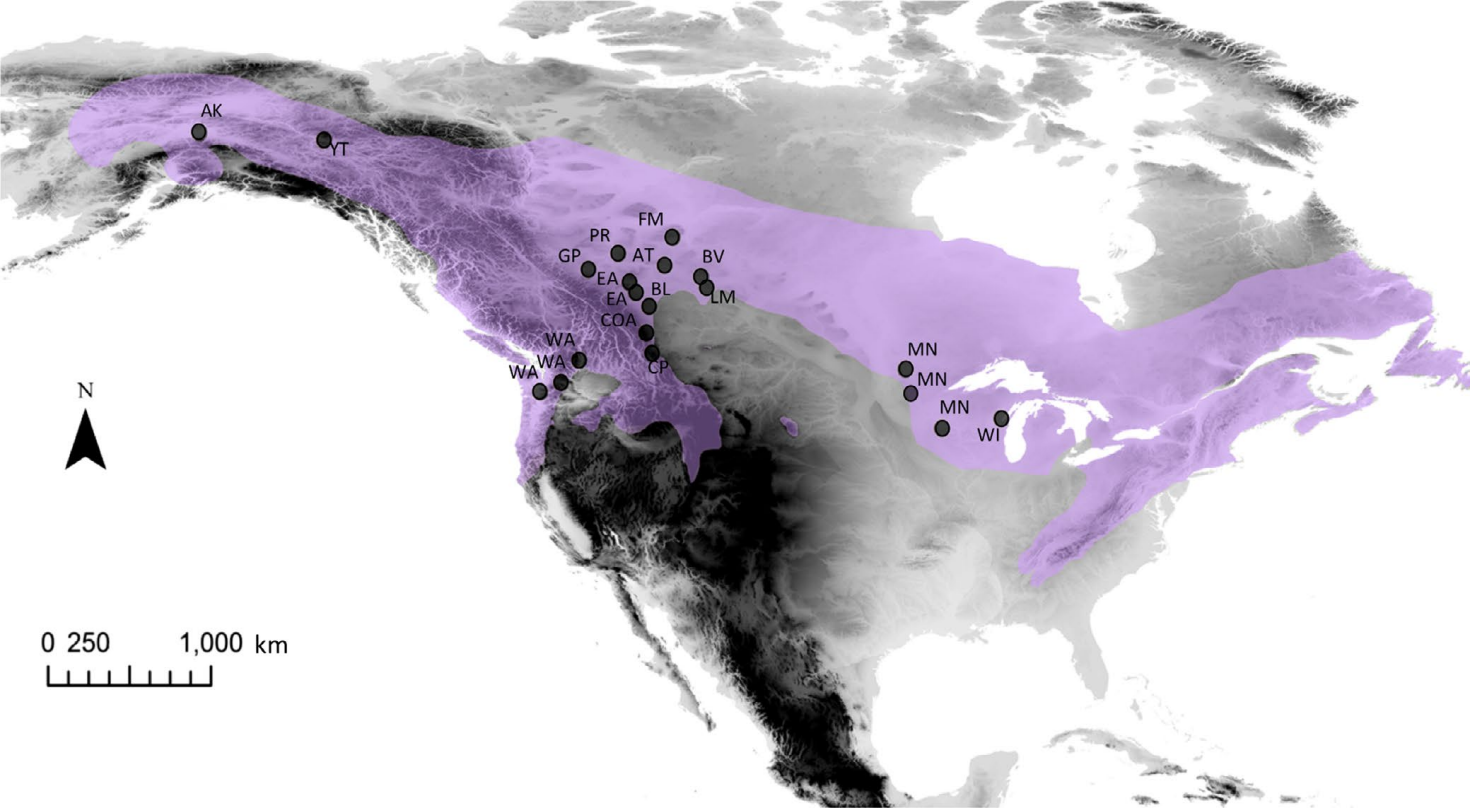
Map showing the current range of Ruffed Grouse (*Bonasa umbellus*), and sampling sites for this study. Sampling sites with the same label were pooled for analyses due to close proximity or lack of sufficient sampling at one or more of these sites. Site abbreviations available in Table 1. The data for the range distribution were taken from Birds of North America Online and were projected and overlaid onto a digital elevation map of North America in ArcGIS® v10.2. Digital elevation map courtesy of ESRI®

The Ruffed Grouse (*Bonasa umbellus*; Figure 1) is widely distributed across North America (Figure 2), is resident throughout its broad distribution, and has relatively low dispersal distances for an avian species (approx. 2–4 km; Yoder, 2004). Furthermore, they inhabit early successional forest, and are closely tied to Quaking Aspen (*Populus tremuloides*), which is an integral part of their diet (Rusch, Destefano, Reynolds, & Lauten, 2000; Svoboda & Gullion, 1972; Zimmerman & Gutiérrez, 2008). Thus, the presence of suitable mixed forest habitat is important for survival, and required for successful dispersal events.

Although Ruffed Grouse have been well studied with respect to ecology and population dynamics (Atwater & Schnell, 1989; Gullion, 1984; Rusch et al., 2000; Zimmerman & Gutierrez, 2007), there is no published information on their population genetics. The Ruffed Grouse is one of the most extensively managed game birds due to heavy hunting pressure throughout most of its range (Rusch et al., 2000). It is also considered an indicator species in the management of early successional forest habitats (USDA Forest Service, 2006). Therefore, information on how macrogeographic barriers and habitat limit gene flow has important implications for managing not only Ruffed Grouse, but also other early successional forest species (e.g., American Woodcock [*Scolopax minor*], Mourning Warbler [*Geothlypis philadelphia*], Golden‐winged Warbler [*Vermivora chrysoptera*]).

The aims of this study were to quantify population structure by assessing gene flow across a large section of the western extent of the species range and to identify geographic barriers and other landscape features that may be restricting or facilitating gene flow. We chose to focus on the western extent of the range because this is where macrogeographic barriers are most likely to be influencing population structure, based on studies in a range of other species (Adams, & Burg, 2015b; Pulgarín‐R & Burg, 2012; Vonhof et al., 2015). Although widespread species with a continuous distribution are expected to show evidence of IBD, we predicted that Ruffed Grouse populations would exhibit patterns of IBR due to the heterogeneous distribution of suitable habitat throughout their range, their low dispersal ability, and their preference for early successional forest habitat. In addition, we predicted that Ruffed Grouse populations would show significant population genetic structuring, and of the extrinsic factors that may be affecting gene flow, both mountains and swaths of unsuitable habitat would be the most likely geographic features to act as barriers.

## 2 MATERIALS AND METHODS

### 2.1 Samples

Fieldwork was conducted from mid‐April through May 2016, during the peak activity period of the male Ruffed Grouse's drumming display (Rusch et al., 2000). Birds were located aurally by drumming activity, and the location of each male's drumming log was marked with a handheld GPS unit. We caught birds with a lift net (Fischer, 1974), a carbon dioxide‐powered net gun, or mirror traps (Gullion, 1965), which were placed on males' drumming logs adjacent to the drumming stage. Brachial venipuncture was used to collect a blood sample, which was stored in 99% ethanol. For this study, we collected 26 Ruffed Grouse samples at two Alberta locations: Buck Lake (52.91 N, 115.01 W), and Crowsnest Pass (49.35 N, 114.40 W). An additional 49 samples were taken from birds collected at the same two sites between 2010 and 2015 for unrelated anatomical studies (Corfield, Harada, & Iwaniuk, 2013; Corfield, Krilow, Vande Ligt, & Iwaniuk, 2013; Krilow & Iwaniuk, 2015). All procedures adhered to the Canada Council for Animal Care regulations were approved by the University of Lethbridge Animal Welfare Committee and collected under research permits issued by Alberta Environment and Parks.

In addition, 159 samples were collected from birds harvested by hunters throughout Alberta in the 2016 hunting season, and 17 were supplied by the Royal Alberta Museum, for a total of 251 samples originating in Alberta (Figure 2). Outside of Alberta, we obtained 100 samples from various western sites with the goal of sampling populations that are likely to be affected by macrogeographic barriers, such as mountain ranges. This included 13 samples supplied by Yukon Fish and Game, 32 from the University of Washington Burke Museum, and 25 from University of Alaska Museum. We also obtained 30 samples in the Great Lakes area from the Field Museum of Natural History to represent a population in the eastern extent of the range. All of the museum samples were collected after 1975.

### 2.2 DNA extraction and amplification

Genomic DNA was isolated from each blood sample with a modified chelex extraction method (Walsh, Metzger, & Higuchi, 1991). Samples were screened at intron 6 of Aldolase B on the Z‐chromosome (Cheviron & Brumfield, 2009), a 394 bp portion of the mitochondrial control region (domain I and II), and an intron of the nuclear *SLC45a2* gene (involved in the melanin production pathway; Gunnarsson et al., 2007). We chose these markers because they are presumed to be neutrally evolving, and this diversity of marker types represents different modes of inheritance and different rates of mutation. Samples were amplified with polymerase chain reaction (PCR) in a 25 µl reaction containing Green GoTaq® Flexi buffer (Promega), 1.5 mM MgCl_2_, 0.08 mM dNTP, 0.4 µM of each primer and 0.5 U GoTaq® Flexi DNA polymerase (Promega) for Aldolase B. These reaction mixes were the same for control region and *SLC45a2*, except for MgCl_2_, for which 2.0 mM was used. Amplification of Aldolase B consisted of one cycle at 95ºC for 120 s, 62°C for 45 s, and 72°C for 60 s; 37 cycles of 94°C for 30 s, 62°C for 45 s, and 72°C for 60 s; followed by a final cycle of 72°C for 300 s. For the control region and *SLC45a2* primers, the annealing temperature was decreased to 54°C. Successfully amplified samples (Aldolase B = 28, and control region = 57, *SLC45a2* = 80) were sequenced at Genome Quebec (Montréal, QC, Canada).

The Aldolase B sequences contained a 7 bp indel. The frequency of the indel differed among populations, so a set of three primers was designed to screen for this indel. The forward primer was placed upstream from the indel, while the other two primers were designed to bind to the insertion and deletion regions, respectively. An M13 tag was added to the 5′ end of the reverse primer for the insertion to increase the size difference between the fragments. Resulting PCR products were 161 bp for the insertion and 118 bp for the deletion. All 351 samples were screened on a 3% agarose gel.

A total of 25 microsatellite loci isolated from species closely related to Ruffed Grouse (Burt et al., 2003; Caizergues, Dubois, Loiseau, Mondor, & Raspluss, 2001; Cheng & Crittenden, 1994; Piertney & Dallas, 1997; Piertney & Hoglund, 2001; Sahlsten, Thörngren, & Höglund, 2008; Segelbacher, Paxton, Steinbruck, Tronteljs, & Storch, 2000; Taylor, Oyler‐McCance, & Quinn, 2003) were optimized and checked for variation. For the 10 polymorphic loci, extracted DNA was amplified in 10 µl reactions containing colorless GoTaq® Flexi buffer (Promega), 2.5 mM MgCl_2_, 0.2 mM dNTP, 0.5 µM of the forward primer, 1.0 µM of the reverse primer, 0.05 µM fluorescent M13 tag, and 0.5 U GoTaq® Flexi DNA polymerase (Promega). LLSD7 and SGCA5 were amplified using 2 mM MgCl_2_ and TTD6 and TUT4 in 2.3 mM MgCl_2_. All forward primers were synthesized with an M13 sequence on the 5′ end of the primer sequence to allow incorporation of the fluorescent M13 tag and visualization of the PCR products on a LI‐COR 4300 DNA Analyzer. To amplify the products, a thermocycling profile with two‐step annealing was used: one cycle of 94°C for 120 s, *T*_A1_°C for 45 s, and 72°C for 60 s; seven cycles of 94°C for 30 s, *T*_A2_°C for 30 s, and 72°C for 45 s; 31 cycles of 94°C for 30 s, 48–62°C for 30 s, and 72°C for 45 s; followed by a final extension step of 72°C for 300 s. Annealing temperatures (*T*_A1_/*T*_A2_) for each primer set differed: TTD2 (45/48); BT18 and TUT4 (48/50); TUT2, BG20, BG15, and TTD6 (52/54); SGCA5 and ADL230 (55/57); and LLSD7 (60/62). PCR products were visualized on a 6% acrylamide gel using a LI‐COR 4300 DNA Analyzer (LI‐COR Inc., Lincoln, NE, USA). Three positive controls of known size were included on each load to ensure consistent scoring. A second person scored all gels to reduce scoring error. As an additional measure against potential errors, a subset of samples from each population were genotyped a second time at each locus.

### 2.3 Genetic diversity analyses

All sequences were checked, manually aligned and assessed for variation using MEGAv6 (Tamura, Stecher, Peterson, Filipski, & Kumar, 2013). DnaSP v5.1 (Rozas, Sánchez‐DelBarrio, Messeguer, & Rozas, 2003) was used to calculate shared haplotypes, nucleotide diversity (*π*), and haplotype diversity (*H*_d_) for control region sequences. The *SLC45a2* sequence data were not variable enough to be informative and were not included in the remainder of our analyses.

Genetic diversity was measured at the population level using microsatellite loci by calculating observed and expected heterozygosities, the number of alleles per locus, and number of private alleles in GenAlEx v6.5 (Peakall & Smouse, 2012). FSTAT v2.3.1.0 (Goudet, 1995) was used to calculate allelic richness (*A*_R_). Genotypes at the microsatellite loci were checked for linkage disequilibrium and deviations from Hardy–Weinberg equilibrium using GENEPOP v4.2 (Raymond & Rousset, 1995) with default parameters. MICRO‐CHECKER v2.2.3 (van Oosterhout, Hutchinson, Wills, & Shipley, 2004) was used to check for errors in the genotyping data including allelic dropout and null alleles. The resulting significance levels were corrected for multiple tests using a modified False Discovery Rate (FDR; Benjamini & Yekutieli, 2001). Two loci, ADL230 and TTD2, were removed due to significant deviation from Hardy–Weinberg equilibrium. The SGCA5 locus had a significant probability of null alleles for a few sampling sites, so analyses were performed with and without this marker to determine if the potential presence of null alleles was biasing the data. SGCA5 was retained in the final analyses because its exclusion did not cause noticeable variation in the results. SGCA5 had more missing data than the other markers (>25% for some sampling sites) and had to be excluded from *F*‐statistic calculations. Of the 351 genotyped samples, 324 were used for analyses after removing samples that amplified at fewer than six loci. Samples collected in the same area on the same day (i.e., hunter‐donated or museum collection samples harvested on the same day) were checked for shared ancestry that would indicate multiple individuals from the same family group; none were found. For analyses that required a priori population assignments, sampling sites within 100 km from each other were grouped as a single “population”. All sampling sites in Washington and all sites in Minnesota were grouped together, respectively, due to low sample sizes at some sites within each state (*n* ≤ 5).

### 2.4 Genetic structure

Genetic differentiation between populations at the control region was determined by calculating Ф_ST_ values in Arlequin v3.5.1.3 (Excoffier, Laval, & Schneider, 2007; Excoffier & Lischer, 2010). *p*‐values were corrected for multiple tests by a modified FDR (Benjamini & Yekutieli, 2001), and control region haplotypes were used to create a statistical parsimony network in PopART v1.7 (Leigh & Bryant, 2015).

Individuals were sexed prior to compiling final genotypes for Aldolase B to determine if each individual was hemizygous (females) or homozygous (males). The allele frequencies were then tested for significant pairwise population differentiation using Fisher's exact test (Fisher, 1922).

Genetic structure was quantified for pairwise comparisons of all populations at microsatellite loci using $F_{\text{ST}}^{\prime }$ (Meirmans & Hedrick, 2011) calculations in GenAlEx v6.5 (Peakall & Smouse, 2012), and *p*‐values were corrected for multiple tests with a modified FDR method (Benjamini & Yekutieli, 2001).

### 2.5 Bayesian clustering analyses

Three Bayesian clustering analyses were performed (STRUCTURE v2.3.4, TESS v2.3, and GENELAND v4.0.6). TESS and GENELAND use geographic coordinates as a parameter for interpreting genetic structure, and the use of multiple Bayesian clustering analyses can help elucidate complex patterns, and aid in validating results (Safner, Miller, McRae, Fortin, & Manel, 2011).

The data were analyzed with STRUCTURE v2.3.4 (Pritchard, Stephens, & Donnelly, 2000) using correlated allele frequencies in the admixture model, and sampling locations as *locpriors*. The *locpriors* option allows sampling location information to be input into the model, but will not create population structuring where there is none. Ten independent runs were performed with 50,000 MCMC repetitions and a 10,000 burn‐in period for *K* values varying from 1 to 10. After these initial runs, values from each *K* for both LnPr(*X*|*K*) and delta *K* (Δ*K*; Evanno, Regnaut, & Goudet, 2005) were averaged in STRUCTURE HARVESTER v0.6.94 (Earl & vonHoldt, 2012) to determine the most likely value of *K* or number of genetic clusters. For the optimal *K*, any clusters that included more than one population were run through the program independently using the same settings to test for additional substructure.

TESS v2.3 (Chen, Durand, Forbes, & François, 2007) was implemented for *K* values from 2 to 10 using 100,000 sweeps and 50,000 burn‐in, and Ψ (value determining how much geographic coordinates influence clustering) was set to 0.6. *K* was selected based on the runs with the highest posterior probability and highest deviance information criterion (DIC). As with STRUCTURE, once *K* was determined, any clusters including more than one population were analyzed independently to test for additional substructure.

GENELAND v4.0.6 (Guillot, Mortier, & Estoup, 2005) was used to evaluate the optimal value of *K* using a correlated alleles model, 500,000 iterations, thinning of 200, a burn‐in of 500, and uncertainty of spatial coordinates set to 10 km. Default settings were used for the maximum rate of the Poisson process, and the maximum number of nuclei in the Poisson–Voronoi tessellation. The most likely value of *K* was determined by examining the posterior probabilities averaged over multiple runs (10 runs, *K* = 1–10), and choosing the *K* value with the highest average posterior probability. Ten additional runs were conducted at this fixed *K* value.

### 2.6 Principal coordinates analysis

To examine genetic structure from a multivariate perspective, we ran a principal coordinates analysis (PCoA) using GenAlEx v6.5 (Peakall & Smouse, 2012). Because it does not make any assumptions about the input data (e.g., Hardy–Weinberg Equilibrium), PCoA is well suited for genetic data (Jombart, Pontier, & Dufour, 2009). Furthermore, patterns revealed by multivariate analyses of genetic data are increasingly being used to further validate Bayesian clustering patterns (Basto et al., 2016). The PCoA analysis was conducted on the matrix of $F_{\text{ST}}^{\prime }$ values for the microsatellite data, and the three axes containing the most variation were retained. A three‐dimensional plot was made in R using the 3D Scatter Plot package (R Core Team, 2016) to visualize the first three principal coordinates.

### 2.7 Species distribution modeling

We constructed a species distribution model (SDM) using 53,145 Ruffed Grouse occurrences from the Global Biodiversity Information Facility (GBIF; http://data.gbif.org/). Observations from nonscientific institutions that were not reviewed or moderated were removed, and we further excluded any occurrences prior to 1980 to ensure accuracy of georeferencing. Environmental data were obtained from the WORLDCLIM dataset (v1.4, http://www.worldclim.org/) at a resolution of 2.5 min, and we used the 19 variables in the BIOCLIM layers (Hijmans, Cameron, Parra, Jones, & Jarvis, 2005) for the most recent time period (1960–1990). The MODIS‐based Global Land Cover Climatology layer (Broxton et al., 2014) from the USGS Land Cover Institute (https://landcover.usgs.gov/) contains high‐resolution data on global land cover types from 2001 to 2010. We added this to the SDM for more accuracy in predicting suitable habitat for Ruffed Grouse. The BIOCLIM and MODIS layers were clipped to the extent of North America and then projected in World Geodetic System 1984 using ArcMap v10.2 (ESRI®).

Data were prepared for ecological niche modeling using the SDMtoolbox v1.1c (Brown, 2014) for ArcGIS. All duplicate occurrence records were removed and rarified at a resolution of 30 km^2^ to reduce sampling bias toward human settlements and roads; 2,421 occurrences were retained. Due to the similarity of some of the climatic variables used in the layers, we tested for layer autocorrelation at the spatial scale of the North American continent. For pairs of layers that were highly correlated (*R* > 0.70), one of each pair was removed from the model until no correlated pairs remained, so as not to bias the SDM. The remaining 10 BIOCLIM layers (1, 2, 3, 4, 8, 9, 12, 14, 15, and 18) were used along with the MODIS land cover layer, and rarefied occurrence data to create the SDM. The Gaussian kernel density tool in SDMtoolbox was used to create a bias layer that was added to the model to aid in further accounting for anthropomorphic bias (Phillips et al., 2009).

The environmental layers and occurrence data were imported into MaxEnt v3.3.3 (Phillips, Anderson, & Schapire, 2006) along with the Gaussian kernel density bias file to create the SDM. The most appropriate settings were determined using the model selection tool in ENMTools v1.3 (Warren, Glor, & Turelli, 2010), optimal corrected Akaike's information criterion (AIC), area under curve (AUC), and Bayesian information criterion (BIC) values. Settings used were as follows: hinge features only, regularization multiplier = 1, a replicate run type of 10 cross‐validations, maximum number of background points = 10,000, 500 maximum iterations and a 0.00001 convergence threshold. For training the model, 25% of the occurrence points were used and the SDM displayed using the cumulative scale.

### 2.8 Dispersal route analyses

To evaluate whether the intervening landscape matrix leads to population differentiation by influencing dispersal routes and dispersal costs (i.e., IBR), we conducted least cost path (LCP) and least cost corridor (LCC) analyses using SDMtoolbox v1.1c (Brown, 2014) in ArcGIS v10.2 (ESRI®). The SDM was inverted to create a friction layer, and coordinates for each sampling site were entered in decimal degrees. LCPs and LCCs were calculated between each pair of populations using the friction values. LCPs are calculated as the dispersal paths of least resistance between each pair of sampling sites based on the resistance surface. To calculate LCCs, the LCPs were weighted by resistance values based on the friction layer and then categorized using a “percentage of LCP” method with cutoffs for inclusion into high‐, mid‐, and low‐classes set at 5%, 2%, and 1% of the LCP value, respectively. The weighted and categorized LCPs were then summed to create a LCC dispersal network.

### 2.9 Isolation by distance and resistance

We tested for IBD with a Mantel test in GenAlEx v6.5 (Peakall & Smouse, 2012) using pairwise $F_{\text{ST}}^{\prime }$ (Meirmans & Hedrick, 2011) and geographic distance. We calculated geographic distance as a straight line between populations, where lines were kept within the boundaries of the species' geographic distribution. This analysis was also conducted using least cost path distance instead of Euclidean distance. To explicitly test for IBR, we used a similar analysis to IBD. Matrices of genetic distance ($F_{\text{ST}}^{\prime }$) and resistance values were assessed for correlations using a paired Mantel test implemented in GenAlEx v6.5 (Peakall & Smouse, 2012). The matrix of resistance values was created from the LCC map by weighting the distance of each LCC by the resistance values along the corridor. After calculating the Mantel tests for all population pairs, the same tests were performed on subsets of populations to examine patterns at a regional scale (if the number of sampling sites permitted).

Because Mantel tests can be prone to Type I error (Legendre & Fortin, 2010; Legendre, Fortin, & Borcard, 2015), the matrices described above were also analyzed using distance‐based Moran's eigenvector map analysis in the MEMGENE package (Galpern, Peres‐Neto, Polfus, & Manseau, 2014) for R. This method finds Moran's eigenvectors in the spatial data (input as geographic, least cost, or resistance distance) using principal coordinates analysis. Then redundancy analysis is used to select a reduced set of vectors based on their contribution as predictors of the response variable (genetic distance). Vectors are added to the model in a stepwise procedure until there is no further improvement of model fit. This method has relatively low error compared to other methods like the Mantel test, as well as the capability to reveal complex patterns (Griffith & Peres‐Neto, 2006; Legendre & Fortin, 2010; Richardson, Brady, Wang, & Spear, 2016). However, due to the constraints on matrix size that can be analyzed in MEMGENE (P. Galpern, pers. comm.), only the full dataset including all 15 populations could be analyzed using both this package and Mantel tests.

## 3 RESULTS

### 3.1 Genetic diversity

The mitochondrial control region sequences (GenBank MK603980–MK604036) from seven populations showed 11 shared haplotypes and 11 singletons. Haplotype diversity (*H*_d_) ranged from 0.400 (BL, CP) to 0.970 (MN), and *π* values from 0.00102 (CP) to 0.02300 (MN; Table 1).

**Table 1:** Number of Ruffed Grouse samples sequenced (*n*) at each sampling site (ID), number of haplotypes (*H*_n_), haplotype diversity (*H*_d_), and nucleotide diversity for mitochondrial control region sequences

Population	ID	*n*	*H*_n_	*H*_d_	*π*
Alaska	AK	15	5	0.743	0.00517
Yukon	YT	–	–	–	–
Washington	WA	10	3	0.800	0.00609
Crowsnest Pass	CP	5	2	0.400	0.00102
Buck Lake	BL	5	2	0.400	0.00203
Edson area	EA	5	3	0.700	0.00457
Lloydminster	LM	5	3	0.900	0.00609
Minnesota	MN	12	9	0.970	0.02300

Of the 19 microsatellite loci that successfully amplified, nine loci were monomorphic (LLST1, LLSD4, TTD1, TUD1, TUD4, TUT1, TUT3, ADL184, RHT0094) and 10 were polymorphic (LLSD7, TTD2, TTD6, TUT2, TUT4, SGCA5, BG15, BG18, BG20, and ADL230). TTD2 and ADL230 were removed due to significant deviations from Hardy–Weinberg equilibrium. For the eight remaining polymorphic loci, the number of alleles per locus ranged from 5 to 28. Observed heterozygosity across loci ranged from 0.548 (YT) to 0.663 (CP), and expected heterozygosity ranged from 0.590 (YT) to 0.688 (COA; Table 2). Significant deviations from Hardy–Weinberg equilibrium only occurred at more than one locus in two populations: BL and EA. The BL population had a significant heterozygote deficit at TUT4, SGCA5, and BG18, and the EA population at BG15, SGCA5, LLSD7, TUT2, and BG18. Allelic richness (*A*_R_) ranged from 3.29 (AK) to 4.10 (WI; Table 2), and 10 of 15 populations contained private alleles (Table 2). Most populations had one to three private alleles, but BL and BV both had five. Also notable was the high frequency (0.14) of a single private allele for the PR population.

**Table 2:** Sampling site or group of sampling sites used in microsatellite analyses (population), sample size per site (*n*), sampling site abbreviation (ID), number of different alleles occurring at a frequency of ≥ 5% (Na), private alleles (PA), allelic richness, (*A*_R_), observed heterozygosity (Ho), and expected heterozygosity (He)

Population	ID	*n*	Na	PA	*A*_R_	Ho	He
Alaska	AK	22	5.25	0	3.29	0.549	0.606
Yukon	YT	13	5.62	2	3.56	0.548	0.590
Washington	WA	23	5.05	2	3.43	0.562	0.635
Crowsnest Pass	CP	36	6.87	2	3.69	0.663	0.674
Cochrane area	COA	12	5.50	0	3.78	0.575	0.688
Buck Lake	BL	29	7.50	5	3.87	0.651	0.682
Edson area	EA	63	9.25	3	3.81	0.586	0.663
Grande Prairie	GP	18	5.87	1	3.85	0.661	0.675
Peace River	PR	8	5.12	–	–	–	–
Athabasca area	AT	29	7.12	2	3.55	0.598	0.620
Fort McMurray	FM	11	5.62	1	3.68	0.589	0.636
Bonnyville area	BV	19	6.62	5	3.77	0.609	0.631
Lloydminster	LM	13	5.62	0	3.64	0.606	0.646
Minnesota	MN	21	6.88	1	4.06	0.590	0.684
Wisconsin	WI	7	5.38	–	–	–	–

Notes

Statistics that may be sensitive to low sample sizes were excluded for populations where *N* < 10.

### 3.2 Genetic structure

**Figure 3:**
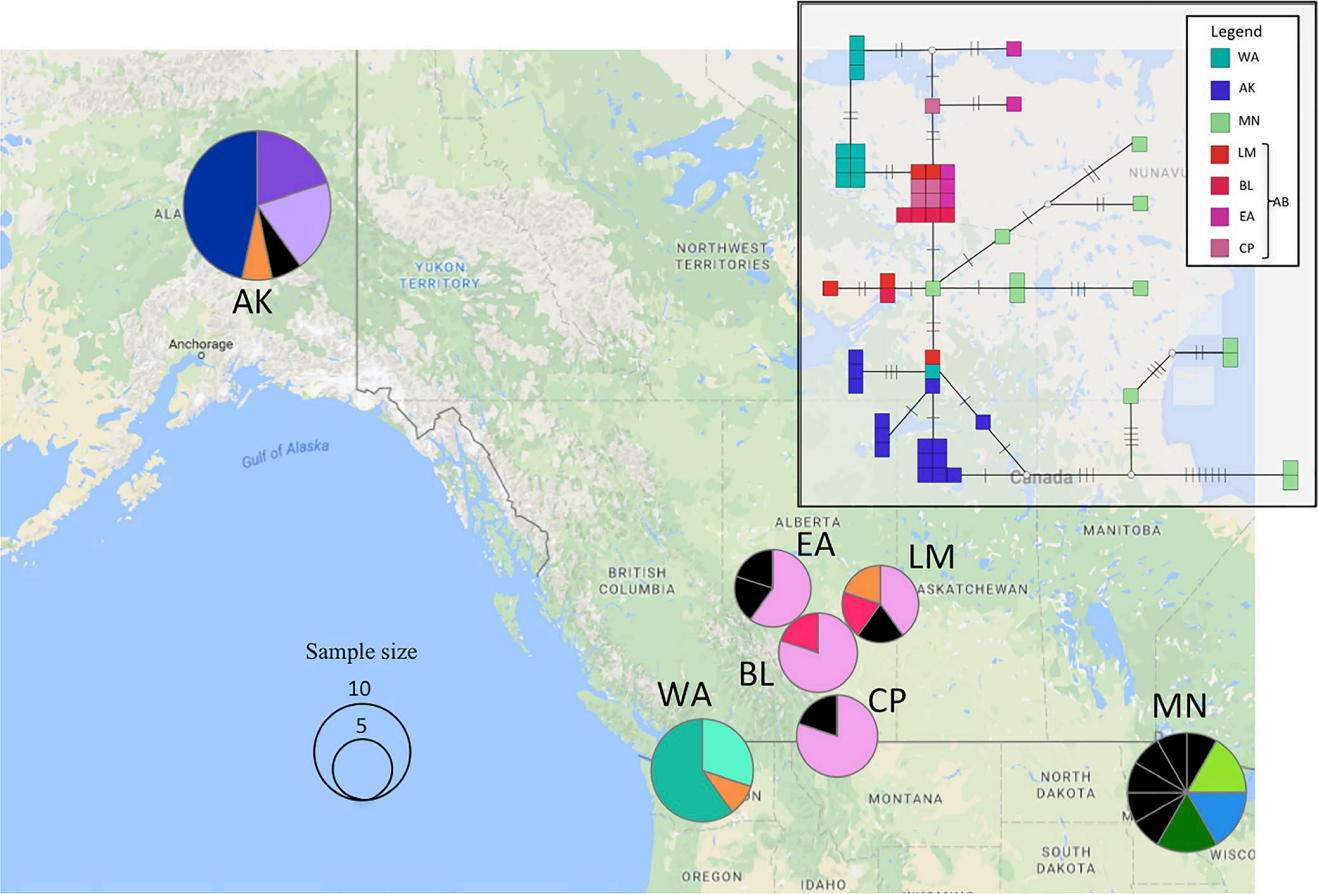
The statistical parsimony network from PopArt v1.7 (Leigh & Bryant, 2015) using control region sequences is shown in the inset. Each individual is a box, and individuals sharing haplotypes are grouped. On the lines that connect haplotypes, each hatch‐mark across a line represents a mutational step, and nodes with inferred haplotypes are denoted by open circles. The geographic distribution of shared haplotypes can be seen on the map. On the map, each haplotype is represented by a different color, singletons are denoted in black, and pie charts are sized based on the number of samples (*n*)

Pairwise Ф_ST_ values for the CR locus ranged from −0.057 for the CP:EA comparison to 0.660 for the AK:WA comparison (Table 3). Furthermore, all 11 significant Ф_ST_values included WA or AK. The statistical parsimony network for CR exhibits noticeable spatial structure (Figure 3). The samples from the Alaska population cluster together within the network, as do most of the samples from Washington. These two populations are the only two groups that are significantly different from other populations according to Ф_ST_values. Samples from Alberta (CP, BL, EA, LM) loosely cluster together on the network, but also share haplotypes with other populations (Figure 3). The Minnesota (MN) samples also show a slight geographic pattern with two clusters, but the most noticeable characteristic of this population is the large diversity of haplotypes present (*H*_d_ = 0.97; Figure 3).

**Table 3:** Pairwise Ф_ST_ values of control region for seven populations of Ruffed Grouse are below the diagonal

	AK	WA	CP	BL	EA	LM	MN
AK	**·**						
WA	**0.660**	**·**					
CP	**0.656**	**0.447**	**·**				
BL	**0.615**	**0.442**	0.000	**·**			
EA	**0.631**	**0.348**	−0.057	0.071	**·**		
LM	**0.450**	**0.431**	0.222	0.000	0.192	**·**	
MN	**0.280**	**0.344**	0.228	0.200	0.212	0.115	**·**

Notes

Comparison values that were significantly different after False Discovery Rate correction are marked in bold.

**Figure 4:**
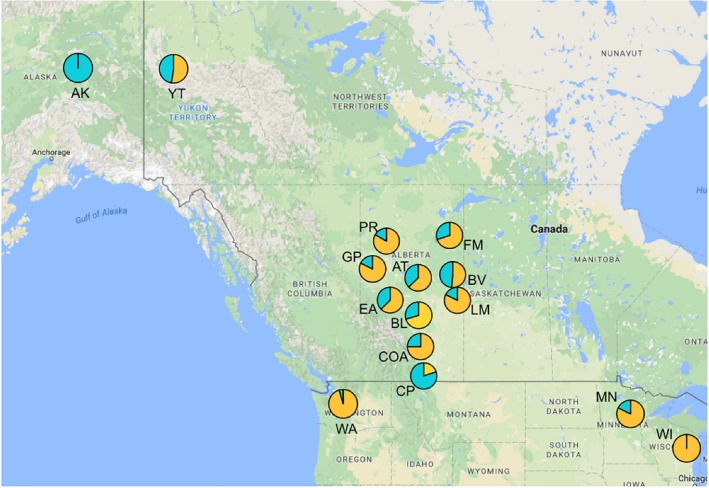
Allele frequencies of a SNP on the Z‐linked Aldolase B gene for Ruffed Grouse from 15 populations. The pie charts show the proportions of the two possible alleles inferred from the screening data at each population. Pairwise comparisons of these population testing for significant differences among the populations can be seen in Table 4

The Fisher's exact tests performed on the Aldolase B SNP resulted in statistically significant comparisons for all population pairs including AK or CP (Table 4; Figure 4). Comparisons between WA and other populations were statistically significant for all but four pairs (COA, GP, PR, and WI). Of all other remaining population comparisons, only three were significant; EA:WI, BV:GP, and BV:WI (Table 4; Figure 4). Like the control region locus, the Aldolase B SNP reveals divergence of the Washington population (Table 4; Figure 4).

**Table 4:** Significance values of Fisher's exact test (Fisher, 1922) for allele frequency pairwise populations comparisons of the biallelic Aldolase B SNP for 15 populations of Ruffed Grouse above the diagonal. Below the diagonal, pairwise F'_ST_ comparisons of data from seven microsatellites

	AK	YT	WA	CP	COA	BL	EA	GP	PR	AT	FM	BV	LM	MN	WI
AK	•	**0.001**	**0.001**	**0.027**	**0.001**	**0.001**	**0.001**	**0.001**	**0.001**	**0.001**	**0.001**	**0.001**	**0.001**	**0.001**	**0.001**
YT	**0.392**	•	**0.001**	**0.025**	0.334	0.316	0.605	0.072	0.253	0.747	0.472	1.000	0.472	0.159	0.032
WA	**0.481**	**0.324**	•	**0.001**	0.060	**0.003**	**0.001**	0.147	0.331	**0.001**	**0.024**	**0.001**	**0.024**	**0.046**	1.000
CP	**0.340**	**0.179**	**0.210**	•	**0.001**	**0.001**	**0.001**	**0.001**	**0.001**	**0.001**	**0.001**	**0.008**	**0.001**	**0.001**	**0.001**
COA	**0.378**	**0.319**	**0.198**	**0.094**	•	0.874	0.472	0.868	0.824	0.566	1.000	0.281	1.000	1.000	0.272
BL	**0.252**	**0.271**	**0.284**	**0.130**	0.033	•	0.472	0.389	0.635	0.586	1.000	0.160	1.000	0.635	0.130
EA	**0.335**	**0.251**	**0.239**	**0.127**	0.008	0.005	•	0.089	0.361	1.000	0.772	0.472	0.772	0.281	**0.041**
GP	**0.288**	**0.242**	**0.193**	**0.097**	−0.016	0.042	**0.027**	•	1.000	0.130	0.472	**0.028**	0.472	0.874	0.472
PR	**0.234**	**0.196**	**0.147**	0.017	−0.001	−0.083	−0.044	−0.028	•	0.472	0.824	0.173	0.824	1.000	0.635
AT	**0.335**	**0.266**	**0.268**	**0.124**	0.098	0.024	0.046	**0.081**	−0.014	•	0.874	0.515	0.874	0.395	0.060
FM	**0.375**	**0.233**	**0.296**	**0.208**	0.045	0.007	0.024	0.096	−0.019	0.022	•	0.403	1.000	0.873	0.158
BV	**0.336**	**0.266**	**0.200**	**0.110**	0.018	−0.013	0.010	**0.065**	−0.060	0.051	0.017	•	0.403	0.100	**0.023**
LM	**0.288**	**0.463**	**0.332**	**0.175**	0.027	0.008	0.058	0.065	−0.016	0.061	0.090	0.016	•	0.873	0.158
MN	**0.387**	**0.292**	**0.186**	**0.203**	**0.206**	**0.225**	**0.164**	**0.067**	0.019	**0.284**	**0.248**	**0.149**	**0.247**	•	0.311
WI	**0.526**	**0.333**	**0.405**	**0.200**	0.079	**0.180**	**0.092**	**0.042**	0.046	**0.235**	**0.174**	**0.146**	**0.210**	0.063	•

Notes

Values that were significant after False Discovery Rate correction for multiple testing are bolded.

**Figure 5:**
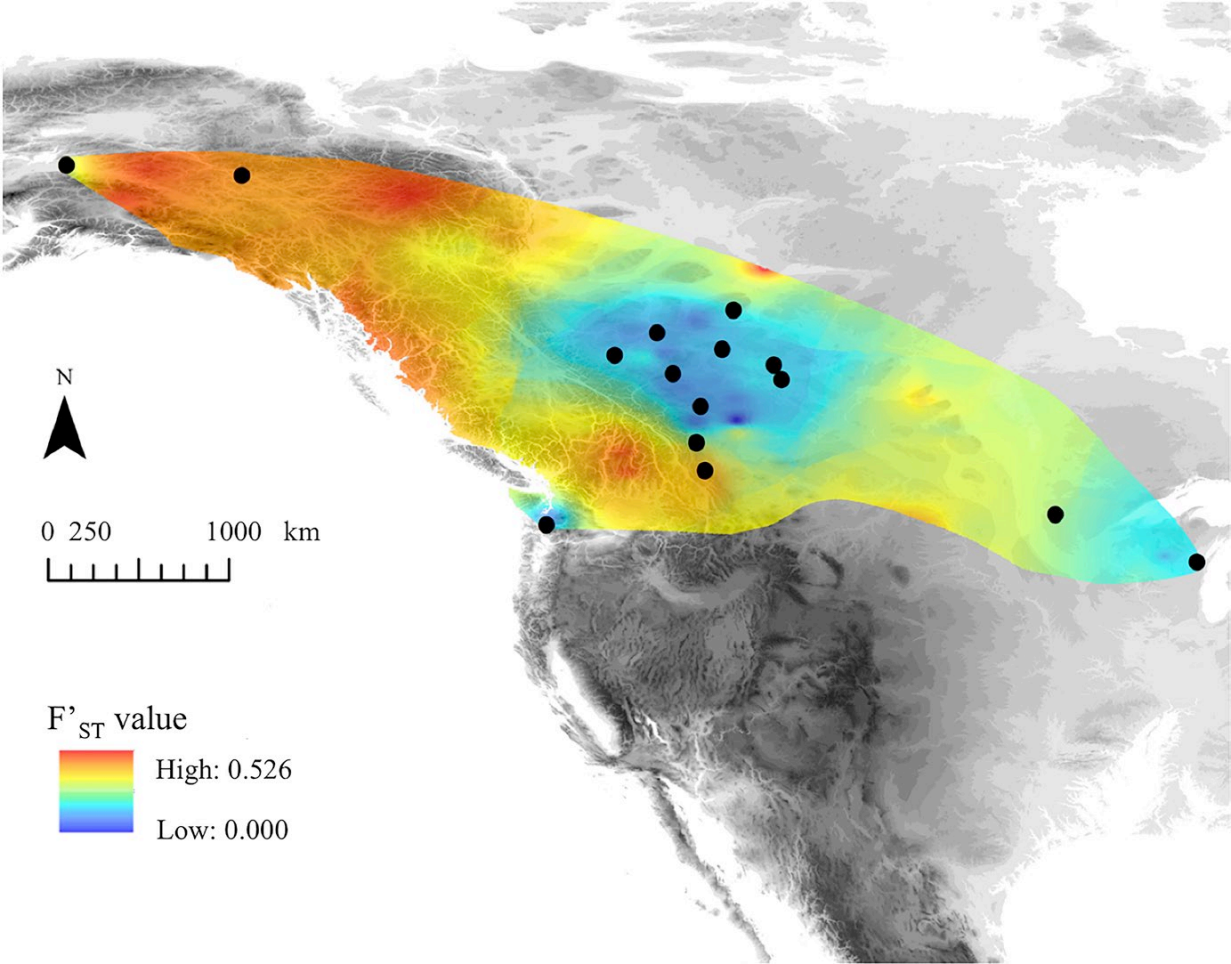
The species divergence map made using the Landscape Genetics toolbox (Vandergast, Perry, Lugo, & Hathaway, 2011) in ArcGIS®. Pairwise $F_{\text{ST}}^{\prime }$ values (Table 4) were color‐coded and interpolated across a geographic map of the sampling sites. Colors that are yellow or warmer are statistically significant $F_{\text{ST}}^{\prime }$ values

Pairwise $F_{\text{ST}}^{\prime }$ values of microsatellite loci ranged from −0.083 (BL:PR) to 0.526 (AK:WI; Table 4). After FDR corrections, 67 out of 105 comparisons were significant. Three populations (AK, YT, and WA) were significantly differentiated from all other populations, while CP was significantly differentiated from all but PR, and MN was differentiated from all but WI and PR. WI was differentiated from all but three populations (MN, PR, and COA). The population divergence map displaying the interpolated pairwise $F_{\text{ST}}^{\prime }$ values clearly shows the low differentiation among all northern and central Alberta populations, and differentiation of the AK, YT, and WA populations (Figure 5).

### 3.3 Bayesian clustering analyses

**Figure 6:**
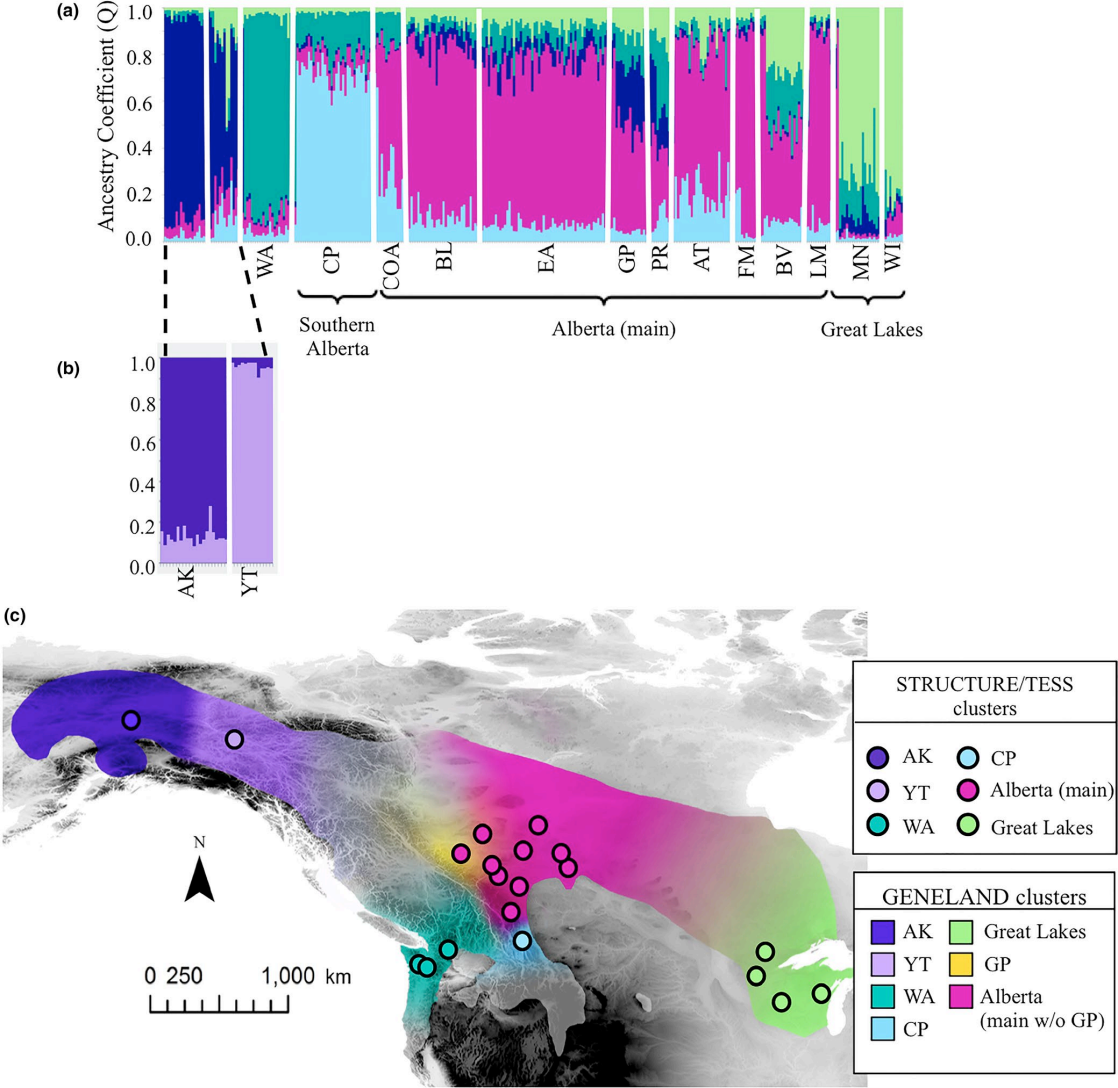
Ruffed Grouse population structure as inferred by hierarchical runs in STRUCTURE v2.3.4 (Pritchard et al., 2000) with eight microsatellite loci. At (a) *K* = 5, and further substructure was apparent when samples from Alaska and Yukon were analyzed independently, which resulted in (b) *K* = 2. No further substructure was found when the remaining clusters were analyzed independently. A (c) map of multiple Bayesian clustering programs where GENELAND clusters have been color‐coded, mapped in geographic space and clipped to limits of the species' range, with the gradient of colors representing clines created by the contour lines of the posterior probability maps in GENELAND (Supporting Information Figure S3). Circles represent sampling sites, and circle color corresponds to the STRUCTURE and TESS consensus cluster assignments. There was only one instance of discordance between the programs: additional cluster (GP) assigned by GENELAND

Plots of delta *K* (Δ*K*) and mean log likelihood (LnPr(*X*|*K*); Supporting Information Figure S1) from the initial STRUCTURE analyses showed *K* = 5. The five clusters were as follows: Alaska + Yukon, Washington, the Great Lakes, Crowsnest Pass, and all remaining sites in Alberta (Figure 6a). To investigate additional population structure, we analyzed each cluster independently. The AK‐YT cluster showed evidence of additional structure (*K* = 2; Figure 6b). Splitting the AK‐YT cluster in two creates a total of six clusters from the STRUCTURE analyses which is concordant with the pairwise $F_{\text{ST}}^{\prime }$ values (Table 4).

Both DIC and log likelihood plots of the Bayesian clustering analysis performed in TESS showed the most likely number of clusters as *K* = 4 with potential substructure (Supporting Information Figure S2). The DIC plot was bimodal with a second peak at *K* = 7 (Supporting Information Figure S2a); however, when examined, the Q plots for *K* = 7 showed clear oversplitting of clusters. We therefore concluded that once hierarchical analysis was performed to reveal substructure, the true number of clusters was *K* = 6 (Supporting Information Figure S3), which is concordant with both STRUCTURE and $F_{\text{ST}}^{\prime }$ results.

The GENELAND analysis showed evidence of *K* = 7 clusters at the highest frequency over the MCMC chain, which was in agreement with the highest value for the averaged posterior probabilities of the initial set of runs. Five of the seven groupings identified by GENELAND corroborated the clusters inferred by STRUCTURE and TESS: AK, YT, WA, CP and Great Lakes. In addition to those five, GENELAND split GP from the remaining Alberta sampling sites (Figure 6c; Supporting Information Figure S4).

### 3.4 Principal coordinates analysis

**Figure 7:**
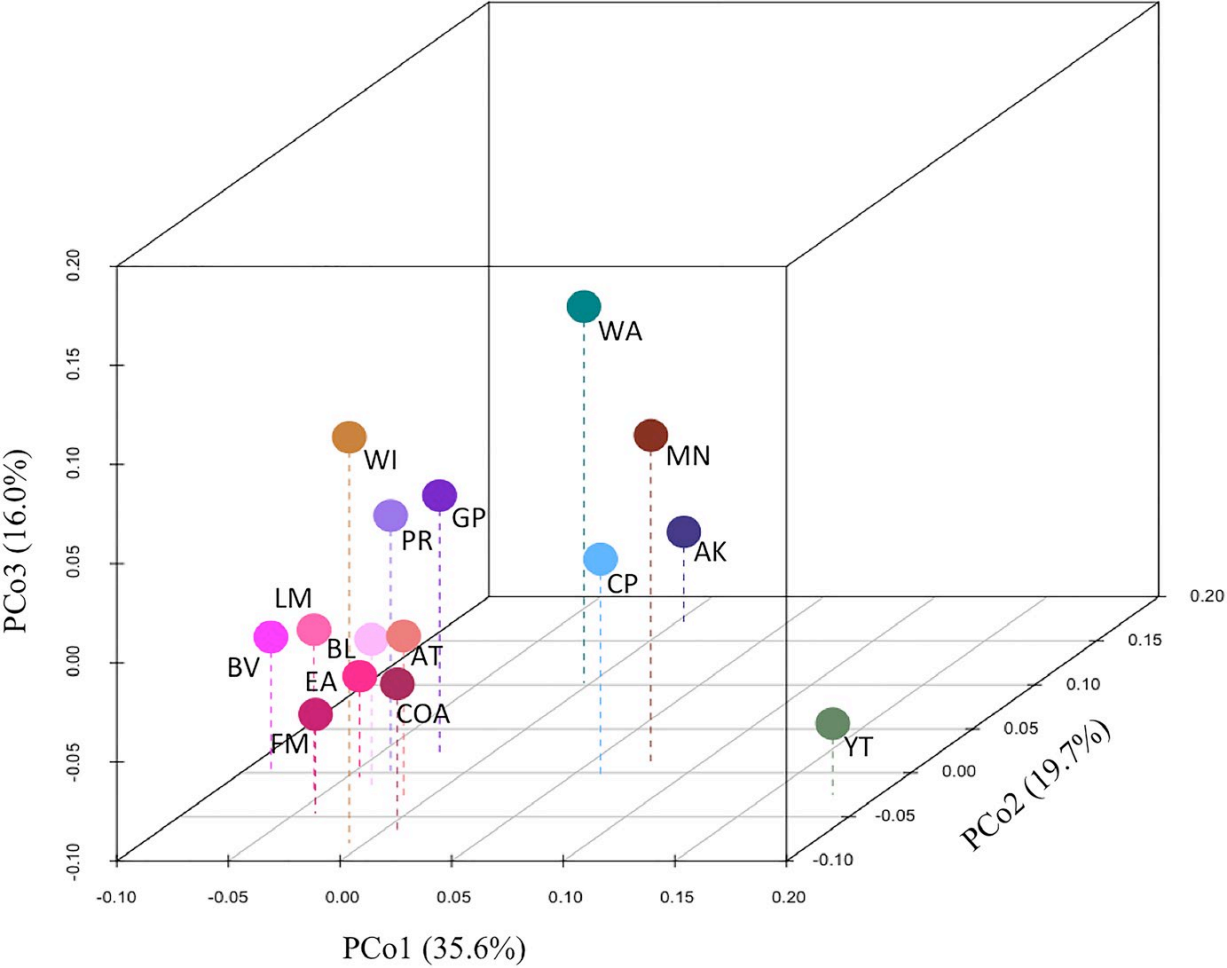
A three‐dimensional plot of the first three axes of the PCoA as calculated in GenAlEx v6.5 (Peakall & Smouse, 2012). Populations are labeled, and principal components are labeled on their respective axes including the amount of variation captured by each in R (R Core Team 2016)

The PCoA using $F_{\text{ST}}^{\prime }$ values showed distinct genetic groupings, with the first and second axes accounting for 35.6% and 19.7% of the variation, respectively, and the third axis explaining 16.0% of the variation. When all three axes are examined together as a three‐dimensional plot, it is clear that AK, YT, WA, CP, MN, and WI are separated from all other populations (Figure 7). The majority of the Alberta sampling sites (COA, BL, EA, AT, FM, BV, LM) cluster together as they do in all other analyses, and the GP and PR sites clustered together. Although GP and PR were separated from other Alberta sampling sites, they were in much closer proximity to these Alberta sites than to the other sampling sites. The groupings of the PCoA confirm groupings identified by TESS and STRUCTURE and provide evidence of GP population divergence from the rest of Alberta as indicated by the GENELAND results (Figure 6b).

### 3.5 Species distribution modeling & dispersal route analyses

**Figure 8:**
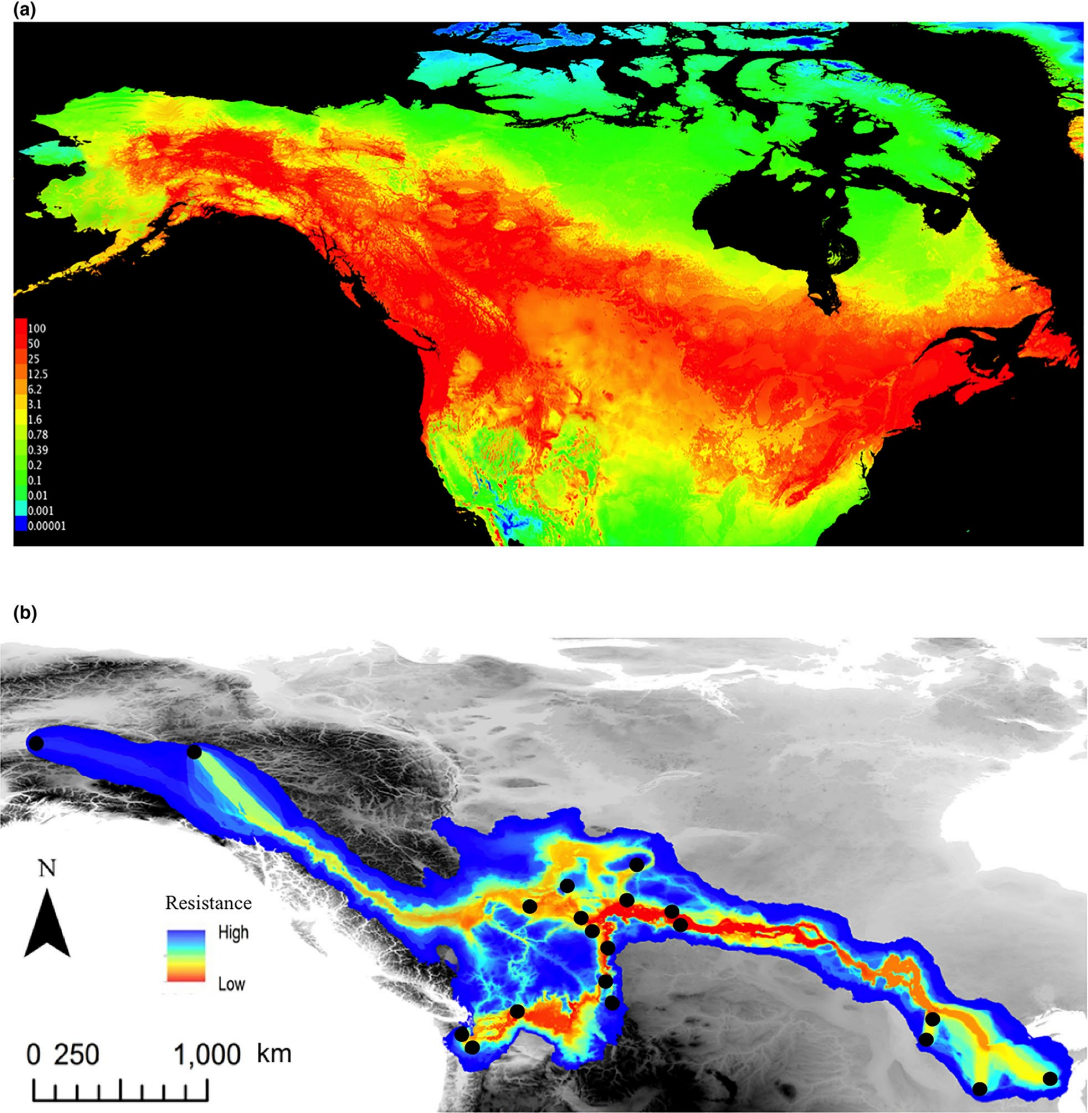
(a) The Species distribution model (SDM) created with SDM toolbox (Brown, 2014) for ArcGIS® and MaxEnt (Phillips et al., 2006). Occurrences from Global Biodiversity Information Facility (GBIF; http://data.gbif.org/) and environmental layers (climate and vegetation data) were input into the model. The SDM shows areas where the environmental conditions are suitable for the Ruffed Grouse to occur (i.e., ecological niche). The scale depicted is cumulative and represents the percent likelihood of habitat suitability for Ruffed Grouse based on the model variables. Using a resistance layer created from inverse of the SDM, (b) least cost corridors (LCC) were calculated among the 15 sampled populations of Ruffed Grouse. The LCC provides more information than least cost paths (Supporting Information Figure S4) and shows the most likely dispersal routes among populations as corridors instead of paths. It also provides dispersal costs along these corridors coded by color; red representing areas where there is low resistance (i.e., low dispersal cost), and blue representing areas of high resistance

The most suitable SDM identified by ENMTools v1.3 (Warren et al., 2010) performed significantly better than expected at random with an AUC of 0.799, where 0.5 is when the fit of the model is no better than random, and as values get closer to one, the model approaches a perfect fit. The SDM approximately matches the species' known distribution (Figure 2) indicating that the environmental variables used to build the model were sufficient to accurately reflect the species' habitat preferences (Figure 8a). The layers that contributed most to the model were land cover, annual mean temperature, and isothermality at 36.1%, 22.2%, and 21.9%, respectively.

When the dispersal routes are examined across the LCC corridor map, it is clear that some population pairs appear to have one or more low‐resistance dispersal routes between them, while for others, the only route revealed by the analysis has relatively high resistance. This pattern could change with the addition of sampling sites in the intervening areas (Figure 8b; Supporting Information Figure S5). The LCC revealed high niche connectivity among most of the Alberta populations, particularly those in the center of the province, and a dispersal route with low‐resistance stretching across the parkland between eastern Alberta and the Great Lakes area (Figure 8b). The LCC (Figure 8b) implies high‐elevation mountains may act as barriers to Ruffed Grouse dispersal. There is low niche suitability in much of the high‐elevation mountains (Figure 8a), with one corridor through the Intermountain West, and another along the Peace River valley, which is the only river valley to penetrate the entire width of the Rocky Mountains (Figure 8b; Cannings, Nelson, & Cannings, 2011). The corridor through the Intermountain West appears to provide connectivity between south‐central Alberta and populations west of the Rockies (e.g., Washington). There is potential for moderate dispersal in Washington, and high dispersal through northeastern Washington, and northern Idaho. The dispersal route connecting the Yukon to populations south of it has moderate to high resistance; it passes between the Rocky and Coast Mountains and then connects with the corridor through the Peace River valley (Figure 8b).

### 3.6 Isolation by distance

**Figure 9:**
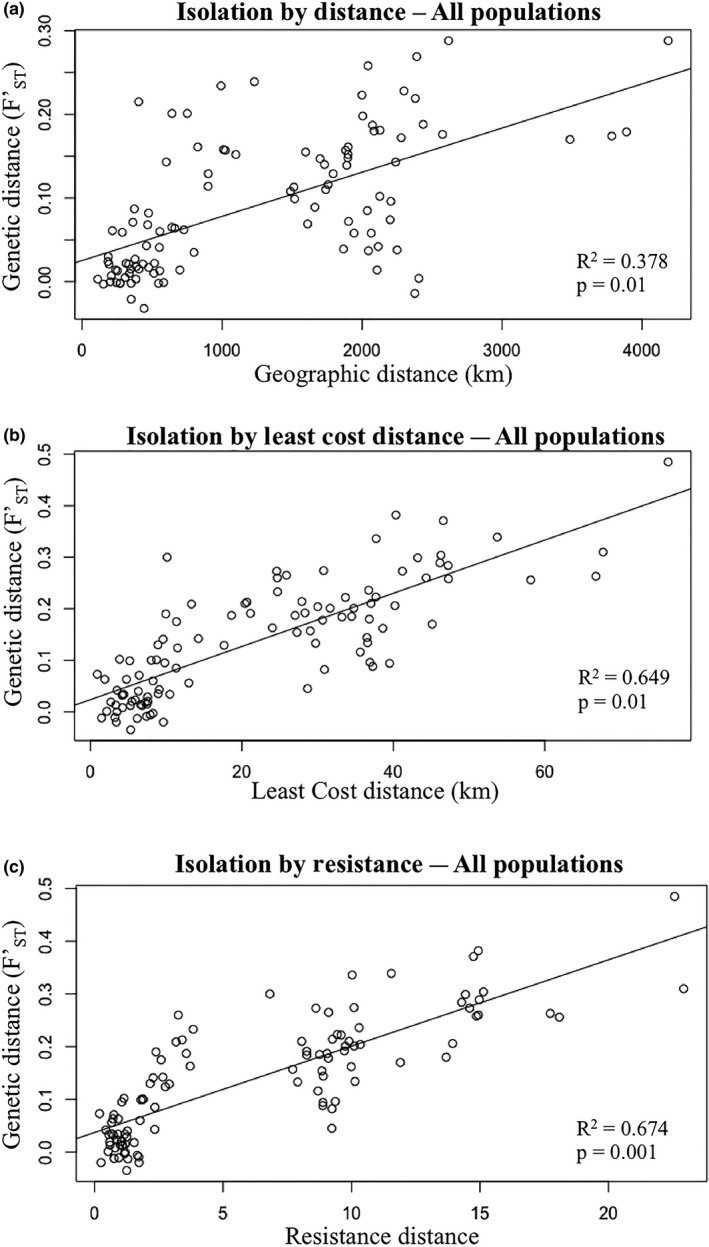
Plots showing Mantel tests of (a) isolation by distance (IBD) comparing genetic distance as measured by $F_{\text{ST}}^{\prime }$ and geographic distance between populations, (b) isolation by least cost distance using least cost paths (LCP), and (c) isolation by resistance (IBR) using resistance‐weighted corridor distances from the LCC map. Correlation values (*R* ^2^) and *p*‐values of each Mantel test are given for each respective plot as calculated in GenAlEx v6.5 (Peakall & Smouse, 2012). Plots shown are comparisons including all 15 sampled populations

The results of the Mantel test for IBD showed a significant correlation between Euclidean distance and pairwise $F_{\text{ST}}^{\prime }$ values when all sampling sites were compared (*R* ^2^ = 0.378; *p* = 0.01; Table 5; Figure 9). MEMGENE results were similar to those of the Mantel test, although a lower proportion of genetic distance was explained by Euclidean distance ($R_{{\mathrm{adj}}^{2}}$ = 0.211; *p* = 0.02; Table 5). When subsets of data were tested for IBD using Mantel tests, only the analysis containing populations east of the Rockies (Alberta and Great Lakes clusters) provided evidence that geographic distance is a significant predictor of pairwise genetic distance (*R* ^2^ = 0.567; *p* = 0.02; Table 5). Analysis including only western populations (WA, AK, and YT) yielded a significant correlation (*R* ^2^ = 0.567, *p* = 0.03), as did the analysis of the Alberta, Alaska, and Yukon clusters (*R* ^2^ = 0.806; *p* = 0.002). The only nonsignificant comparisons are those including CP and remaining Alberta sampling sites (*R* ^2^ = 0.190, *p* = 0.09), and the analysis of sites within the Alberta cluster (COA, BL, EA, GP, PR, AT, FM, BV, LM; *R* ^2^ = 0.082, *p* = 0.063).

**Table 5:** Results of Mantel tests and distance‐based Moran's eigenvector map analysis (dbMEM) for three different models; isolation by distance (IBD), isolation by distance using least cost path distance (LCP), and isolation by resistance (IBR)

Populations compared	IBD	LCP	IBR
Mantel tests			
Overall	*R* ^2^ = 0.370	*R* ^2^ = 0.649	*R* ^2^ = 0.674
	*p* = 0.010	*p* = 0.010	*p* = 0.001
Alberta and Great Lakes (COA, BL, EA, GP, PR, AT, FM, BV, LM, MN, WI)	*R* ^2^ = 0.567	*R* ^2^ = 0.585	*R* ^2^ = 0.655
	*p* = 0.020	*p* = 0.024	*p* = 0.014
Alaska, Yukon, Washington, and S. Alberta (AK, YT, WA, CP)	*R* ^2^ = 0.380	*R* ^2^ = 0.425	*R* ^2^ = 0.579
	*p* = 0.020	*p* = 0.004	*p* = 0.001
Alaska, Yukon, and Washington (AK, YT, WA)	*R* ^2^ = 0.567	*R* ^2^ = 0.668	*R* ^2^ = 0.834
	*p* = 0.030	*p* = 0.042	*p* = 0.019
Alberta, Alaska, and Yukon (COA, BL, EA, GP, PR, AT, FM, BV, LM, AK, YT)	*R* ^2^ = 0.806	*R* ^2^ = 0.835	*R* ^2^ = 0.853
	*p* = 0.002	*p* = 0.001	*p* = 0.004
Alberta, S. Alberta, and Washington (COA, BL, EA, GP, PR, AT, FM, BV, LM, CP, WA)	*R* ^2^ = 0.575	*R* ^2^ = 0.592	*R* ^2^ = 0.645
	*p* = 0.001	*p* = 0.001	*p* = 0.002
Alberta and Washington (COA, BL, EA, GP, PR, AT, FM, BV, LM, WA)	*R* ^2^ = 0.594	*R* ^2^ = 0.631	*R* ^2^ = 0.708
	*p* = 0.010	*p* = 0.010	*p* = 0.020
Alberta and S. Alberta (COA, BL, EA, GP, PR, AT, FM, BV, LM, CP)	*R* ^2^ = 0.190	*R* ^2^ = 0.111	*R* ^2^ = 0.361
	*p* = 0.090	*p* = 0.050	*p* = 0.030
Alberta (within cluster comparison) (COA, BL, EA, GP, PR, AT, FM, BV, LM)	*R* ^2^ = 0.082	*R* ^2^ = 0.065	*R* ^2^ = 0.266
	*p* = 0.063	*p* = 0.065	*p* = 0.267
Distance‐based Moran's Eigenvector Map analysis			
Overall	$R_{{\text{adj}}^{2}}$ = 0.211	$R_{{\text{adj}}^{2}}$ = 0.189	$R_{{\text{adj}}^{2}}$ = 0.487
	*p* = 0.020	*p* = 0.010	*p* = 0.005

Notes

To examine multiple spatial scales, an overall correlation was run for all sampling sites (both Mantel tests and dbMEM), as well as subsets of the different sampling sites (Mantel tests only). The correlation value for each comparison is reported (*R* ^2^ or adjusted *R* ^2^), along with the significance level of each test.

### 3.7 Isolation by resistance

When a Mantel test was performed to test the correlation between LCP distance and genetic distance ($F_{\text{ST}}^{\prime }$), the correlation was considerably higher (*R* ^2^ = 0.649, *p* = 0.01; Table 5) than the value calculated for IBD (*R* ^2^ = 0.370, *p* = 0.01). The correlation between resistance distance and genetic distance was higher yet (*R* ^2^ = 0.674, *p* = 0.001). MEMGENE analysis using least cost distances provided an adjusted *R* ^2^ value ($R_{{\text{adj}}^{2}}$ = 0.189; *p* = 0.01; Table 5) similar to that of the IBD analysis. However, using resistance distances, both the MEMEGENE and Mantel tests were similar and accounted for considerably more of the variation in the genetic data than the other two distance measures ($R_{{\text{adj}}^{2}}$ = 0.487; *p* = 0.005; Table 5). When Mantel tests were performed on subsets of populations, the IBR model explained more of the variation in genetic data than either the IBD or LCP models for all subsets (Table 5). The only group that did not yield a significant correlation were the Alberta populations (excluding CP). No other groups had enough sampling sites to perform within region comparisons.

## 4 DISCUSSION

Using multi‐locus genetic data and environmental variables, we found significant genetic differentiation and limited connectivity among western populations of Ruffed Grouse. Macrogeographic barriers, tracts of unsuitable habitat, and the species' preference for aspen‐dominated mixed forest are likely playing important roles in creating genetically structured populations.

### 4.1 Contemporary population genetic structure and macrogeographic barriers

Data from multiple neutral genetic markers show structuring of Ruffed Grouse populations across their range at multiple spatial scales. Aside from the most highly differentiated populations, AK and WA, at least four other distinct genetic groups exist: Yukon, southwest Alberta (CP), a large group including most of central/northern Alberta, and one group near the Great Lakes (Figure 6b). Although these results support our hypothesis of genetic differentiation due to low dispersal ability, the degree of differentiation is somewhat unexpected.

A number of landscape features co‐occur with the boundaries of genetic clusters for Ruffed Grouse across western North America. The Columbia River basin (southeast of WA sites) and northern extent of the Great Plains (southeast of Alberta sites and west of the Great Lakes) impose sharp limits on the species' range (Figure 2) and mountain ranges appear to be a prominent barrier within the western extent of the range. The Alaska, Wrangell, Ray, and Chugach Mountains effectively isolate the Alaska population, as supported by divergence of this population at the microsatellite loci, Z‐linked SNP, and control region. In addition, the mitochondrial control region shows very little haplotype sharing with any of the other sampling sites (Figure 3). The Yukon population is isolated by the same mountain ranges preventing connectivity with the Alaska population, and by the Mackenzie Range potentially restricting connectivity with populations to the east. Mountains also co‐occur with genetically restricted populations in other parts of the range; a genetic break is present between the Washington population and the Alberta populations suggesting restricted movement across the Rocky Mountains. The Washington population is genetically distinct, as supported by the microsatellite data (Table 4), Z‐linked marker data (Table 4), and minimal haplotype sharing in the control region (Figure 3). If the Cascade Range is acting as a barrier, substructure should have been detected within the WA cluster through Bayesian analyses because the sampling sites were on both sides of the Cascades. Bayesian methods do not use a priori population assignments, so any potential substructure should be apparent in the analyses, regardless of how samples were grouped (Figure 6a,b; Supporting Information Figures S3 and S4). The Cascade Range contains more suitable mixed forest habitat (Broxton et al., 2014; Pater, Bryce, Thorson, Kagan, & Chapell, 1998), and generally lower elevation passes than the northern Rockies (Franklin & Dyrness, 1973). Similar patterns of differentiation occur in a widespread generalist passerine, the Black‐capped Chickadee (*Poecile atricapillus*), where the habitat composition of a mountain range corresponds to restricted gene flow (Adams, & Burg, 2015b). The Black‐capped Chickadee also has similar patterns of isolation in Alaska, northwest British Columbia, and on either side of the southern Rockies (Adams, & Burg, 2015b; Hindley, Graham, & Burg, 2018).

The increased number of sampling sites in Alberta allowed us to assess genetic structure on a finer scale. While most of the Alberta populations are not differentiated from one another, evidence from the nuclear loci show that the Crowsnest Pass population is isolated from all other populations (Figures 4, 5 and 6b). Because CP does not show significant differentiation at the control region (Figure 3), it is unlikely that divergence of this population reflects historical isolation. Instead, the differentiation of the CP population likely arose due to contemporary barriers to gene flow. In some species, southwest Alberta populations are divergent from individuals sampled throughout the rest of Alberta, and instead group with either British Columbia populations (Adams, & Burg, 2015a) or Intermountain West (i.e., Montana, Idaho, Wyoming) populations (Dohms, Graham, & Burg, 2017; Pulgarín‐R & Burg, 2012) implying that the geography of the Rocky Mountains may affect the genetic structure of species differently depending on their life history.

The presence of unsuitable habitat may also be restricting gene flow, particularly for the CP population. Only a narrow swath of suitable Ruffed Grouse habitat presently connects southwestern Alberta and the rest of the province; most of the southeast part of the province is open grassland, which this species is reluctant to disperse through (Yoder, 2004), and the Rocky Mountains run along the western edge of the province. The habitat in the Rocky Mountains consists mainly of contiguous coniferous forest, with suitable mixed forest habitat occurring mostly on low elevation slopes and valleys (Broxton et al., 2014; Natural Regions Committee, 2006). Although Ruffed Grouse are more likely to disperse through coniferous forests than grasslands, their short dispersal distances (approx. 2–4 km; Yoder, 2004) suggest that dispersal through vast expanses of coniferous forest are likely to be infrequent. Because the CP population is in close spatial proximity to some of the other populations sampled in Alberta, geographic distance is unlikely to be a causal factor for population differentiation and this is corroborated by IBD analysis (Table 5). Therefore, the combination of the Rocky Mountains as a physical barrier, as well as the northwest corner of the Great Plains (where they meet the Rocky Mountains) are likely the main factors isolating the CP population. However, this assertion would be strengthened by the addition of sampling locations west of the Rockies, such as sites in Montana, Idaho, and British Columbia.

While there is not complete consensus across our analyses for the GP cluster, there is certainly evidence of differentiation of this population, which could be due to its proximity to the Peace River valley. The Peace River is the only river to cut a continuous valley through the entire width of the Rocky Mountain range (Feinstein, 2010). It is possible that genotype frequencies at GP are subject to an influx of genes from British Columbia through the Peace River valley corridor, a low elevation valley through the Rocky Mountains. This is supported by the STRUCTURE results, in which PR and GP show some admixture with the AK/YT cluster (Figure 6a). The Peace River valley may be a contact zone for Ruffed Grouse populations on either side of the Rocky Mountains. There is evidence that this important corridor facilitates connectivity for multiple species that are otherwise geographically isolated by the Rockies, particularly those reliant on mixed forest or shrubby habitats, and those that would have difficulty dispersing through coniferous forests (Irwin, Brelsford, Toews, MacDonald, & Phinney, 2009; Seneviratne, Davidson, Martin, & Irwin, 2016; Toews, Brelsford, & Irwin, 2011). Furthermore, the permeability of a mountain barrier may range from porous (Vonhof et al., 2015) to impermeable (Irwin, Irwin, & Smith, 2011) depending on the species. Sampling of Ruffed Grouse in British Columbia is required to further test the extent to which the northern Rocky Mountains are a permeable barrier.

### 4.2 Landscape genetics: isolation by distance or resistance?

Aside from mountain ranges, the presence of unsuitable habitat is the most prevalent potential barrier between our sampling sites. Due to the seemingly high degree of habitat heterogeneity across the landscape, we incorporated environmental variables into our analyses to test their effects on the genetic structuring present, and to help further test the presence of putative geographic barriers discussed previously. LCC analysis revealed that dispersal cost varies across the landscape and confirms our earlier hypothesis that mountain ranges are likely to impede dispersal among populations. Mountain ranges in Alaska, as well as the Rockies have markedly high levels of resistance to Ruffed Grouse dispersal, with the exception of two corridors through the mountains: one through the Peace River valley, and one through the Intermountain West, connecting south‐central Alberta and northeast Washington (Figure 8b). These corridors are lower elevation areas with slightly milder climate and a higher proportion of mixed forest than the surrounding mountain slopes (Broxton et al., 2014; Hijmans et al., 2005). No direct dispersal routes could be identified among Yukon, Alaska, and Washington populations, and the only dispersal route connecting Alaska to the other sampled populations has high resistance. It should, however, be noted that the lack of sampling from within British Columbia may have prevented the identification of dispersal routes among these population. That said, the LCCs occurred mainly in areas with tracts of mesic, mixed forest, which implies that variation in climate and forest type across the landscape may be important in creating population structuring.

Across all populations, IBR explained significantly more of the genetic differentiation than IBD at most spatial scales (Table 5); the only exception being the comparisons within Alberta. The LCC between Alberta and the Great Lakes had low resistance, stretching across the parkland/boreal forest in a direct path (Figure 8b). Because much of the landscape between these two regions presents low dispersal resistance, it is not unusual that IBR only moderately outperformed IBD. This implies that the genetic differences between Alberta and Great Lakes populations are explained by a combination of physical distance and dispersal cost through intervening habitat at this large spatial scale, although this should be verified further by sampling individuals in between these two populations (e.g., Saskatchewan, Manitoba). In contrast, the genetic differentiation among western populations (AK, YT, WA) was better explained by IBR than by IBD alone (Table 5). This is concordant with the LCP/LCC maps, which do not show any direct dispersal routes between the AK, YT, and WA populations. Furthermore, the routes that were detected have moderate to high resistance. The patterns lend support to the idea that the higher heterogeneity of habitat types west of the Rocky Mountains are restricting dispersal, but sampling of BC populations is required to rule out the effects of small sample sizes.

Due to large gaps among our sampling sites outside of Alberta, it is difficult to be certain whether genetic boundaries between populations are gradual genetic clines or genetic breaks. IBD manifests as smooth, clinal gradients between genetic clusters (Mims et al., 2016), whereas distinct boundaries among genetic clusters are more likely to occur in populations mediated by IBR (Coulon et al., 2006). The high pairwise $F_{\text{ST}}^{\prime }$ values and relatively steep genetic cluster boundaries (Table 4; Figure 5; Supporting Information Figure S3) provide further evidence that Ruffed Grouse populations are distinct genetic clusters mediated by IBR. Furthermore, the patterns of IBR among Alberta populations and those west of the Rockies point to the Rocky Mountain range as a barrier. The conifer‐dominated habitat characterizing high‐elevation mountains has high dispersal costs and would explain patterns of reduced connectivity in Ruffed Grouse found in the SDM and LCC (Figure 8a,b).

Although IBR performed consistently better than IBD, both of these models only explained a small to moderate proportion of the genetic differentiation between CP and adjacent populations (Table 5). CP might therefore have additional factors affecting connectivity with other Ruffed Grouse populations. The environmental variables used in the LCC analysis explained much of the differentiation present among the populations sampled for this study (Table 5), but it is possible that additional, unsampled environmental factors are contributing to the genetic break at the CP population. In some species, genetic structure may not be evident at a broad spatial scale and is only detected when populations are assessed at a finer scale (Adams, & Burg, 2015a). Sampling in other areas such as British Columbia, Saskatchewan, and Manitoba could also provide additional insight into Ruffed Grouse population structure. Therefore, a smaller scale analysis with more environmental variables and higher resolution sampling may aid in identifying the factors underlying the genetic differentiation of the CP population. Furthermore, differences in the vegetation communities of the Intermountain West (including southwestern Alberta) compared to the boreal region (including central‐northern Alberta) may be important. The shift in *Populus* species (e.g., *P. trichocarpa*, *P. angustifolia*, *P. balsamifera*) between these regions is an example of how the environmental conditions in these areas differ and may be contributing to genetic structure through local adaptation. These shifts in vegetation communities correspond to patterns of genetic variation in other avian species in the Intermountain West and central Alberta (Adams, & Burg, 2015a; Dohms et al., 2017).

## 5 CONCLUSIONS

Our study is a first look into the population genetics of Ruffed Grouse, and we found that contemporary populations in the western extent of the range were highly genetically structured, with the strongest genetic breaks co‐occurring with high‐elevation mountain ranges and habitat. Resistance modeling revealed genetic structure in Ruffed Grouse is primarily influenced by the heterogeneous habitat mosaic of the western North American landscape. Dispersal routes appear to be restricted to areas with mixed forest habitat supporting our hypothesis of IBR in Ruffed Grouse, despite its widespread and relatively continuous distribution. In doing so, this study represents one of relatively few contemporary population genetic studies focusing on broadly distributed organisms, and one of even fewer revealing patterns of IBR in widespread, fairly continuously distributed organisms that may be expected to exhibit IBD (Ball, Finnegan, Manseau, & Wilson, 2010; Pease et al., 2009; Pilot et al., 2006). As such, we should no longer assume that dispersal distance and distribution are the only factors driving dispersal patterns, and that IBR is only likely to affect species at small scales. This adds to the growing body of work highlighting the importance of evaluating the role of environmental variables in population genetic structuring (McRae & Beier, 2007; Vergara et al., 2015). It also underscores the need for more landscape genetic studies focusing on broadly distributed taxa because they may be experiencing genetic isolation regardless of their relatively ubiquitous distributions.

## CONFLICT OF INTEREST

None declared.

## AUTHOR CONTRIBUTIONS

AMJ, TMB, and ANI designed the study; AMJ, NPO, and ANI conducted fieldwork and obtained samples; AMJ performed all laboratory work and analyses with help from TMB; AMJ, TMB, and ANI interpreted the results; AMJ, TMB, and ANI prepared the manuscript. All authors revised, read, and approved the final manuscript.

## Data Availability

Genotype data available from the Dryad Digital Repository: https://doi.org/10.5061/dryad.p353tk1, and DNA sequences are available through GenBank (MK603980–MK604036).
