# Supplementary material for: Landscape effects on the contemporary genetic structure of Ruffed Grouse (Bonasa umbellus) populations
Source: Ecol Evol. 2019 May 1;9(10):5572–92. doi: 10.1002/ece3.5112 (PMC6540679; doi:10.1002/ece3.5112)
Supplement: Supplementary file 1 [file ECE3-9-5572-s001.docx]

**APPENDICES**

a)

b)

**Figure S1.** Plots of (a) log likelihood (LnPr(X|K)) and (b) delta K (∆K) over 10 replicates of STRUCTURE analyzed for each value of K from 1-10. Plots were created using STRUCTURE HARVESTER v0.6.94 (Earl and vonHoldt 2012), and were used to evaluate all 15 populations run together; subsequent runs involving only two populations to investigate substructure cannot be plotted. The most likely number of populations (K) is determined by the maximum estimated log probability of the data and highest delta K.


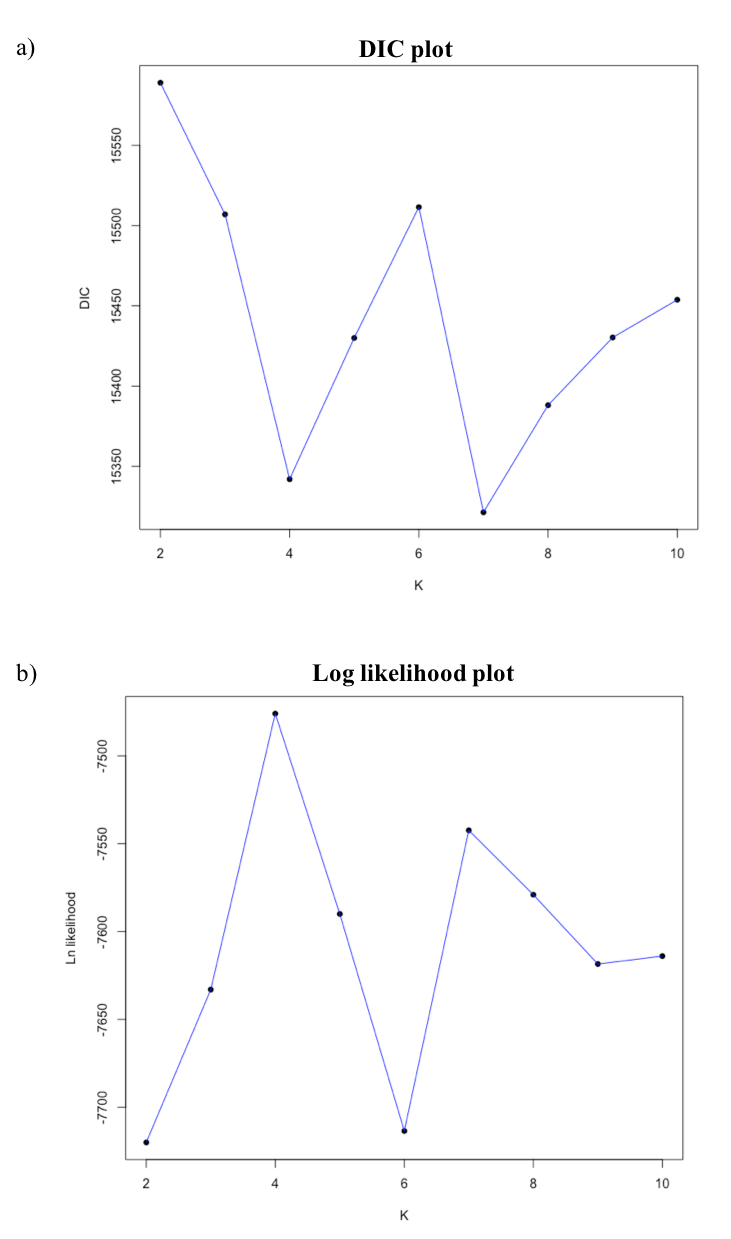


**Figure S2.** Plots of (a) DIC and (b) log likelihood averaged over 10 runs in TESS v2.3 from K = 2 – 10. The lowest value of DIC suggests the best value of K, whereas the highest value of log likelihood (LnPr(X|K)) indicates the most appropriate value of K.


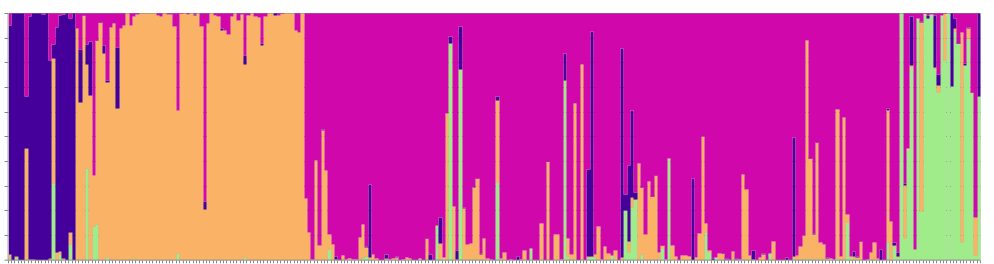

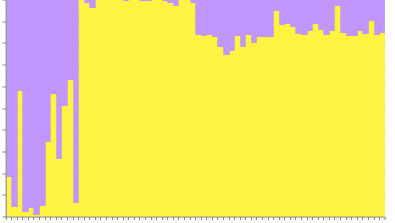

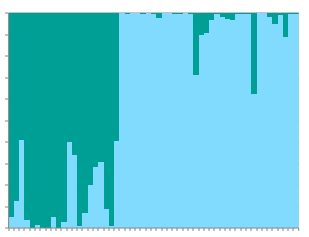

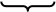


0.4

1.0

0.8

0.6

0.2

0.0

AK

LM

BV

FM

AT

PR

GP

EA

BL

MN

WI

YT

WA

CP

Alberta (main)

Great Lakes


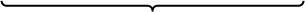


COA

a)

b)

c)

**Figure S3.** Ruffed Grouse population structure as inferred by TESS v2.3 (Chen et al. 2007) from eight microsatellite loci. The histogram plots show ancestry coefficient (Q) on the y-axis, and individual samples on the x-axis. The number of genetic cluster and inferred membership to these clusters for (a) K = 4. Further substructure was identified by running the cluster containing YT, WA, and CP independently at (b) K = 2, and after removing YT, populations WA and CP were run at (c) K = 2. No further substructure was found when the remaining clusters were run independently.

**a)**

**
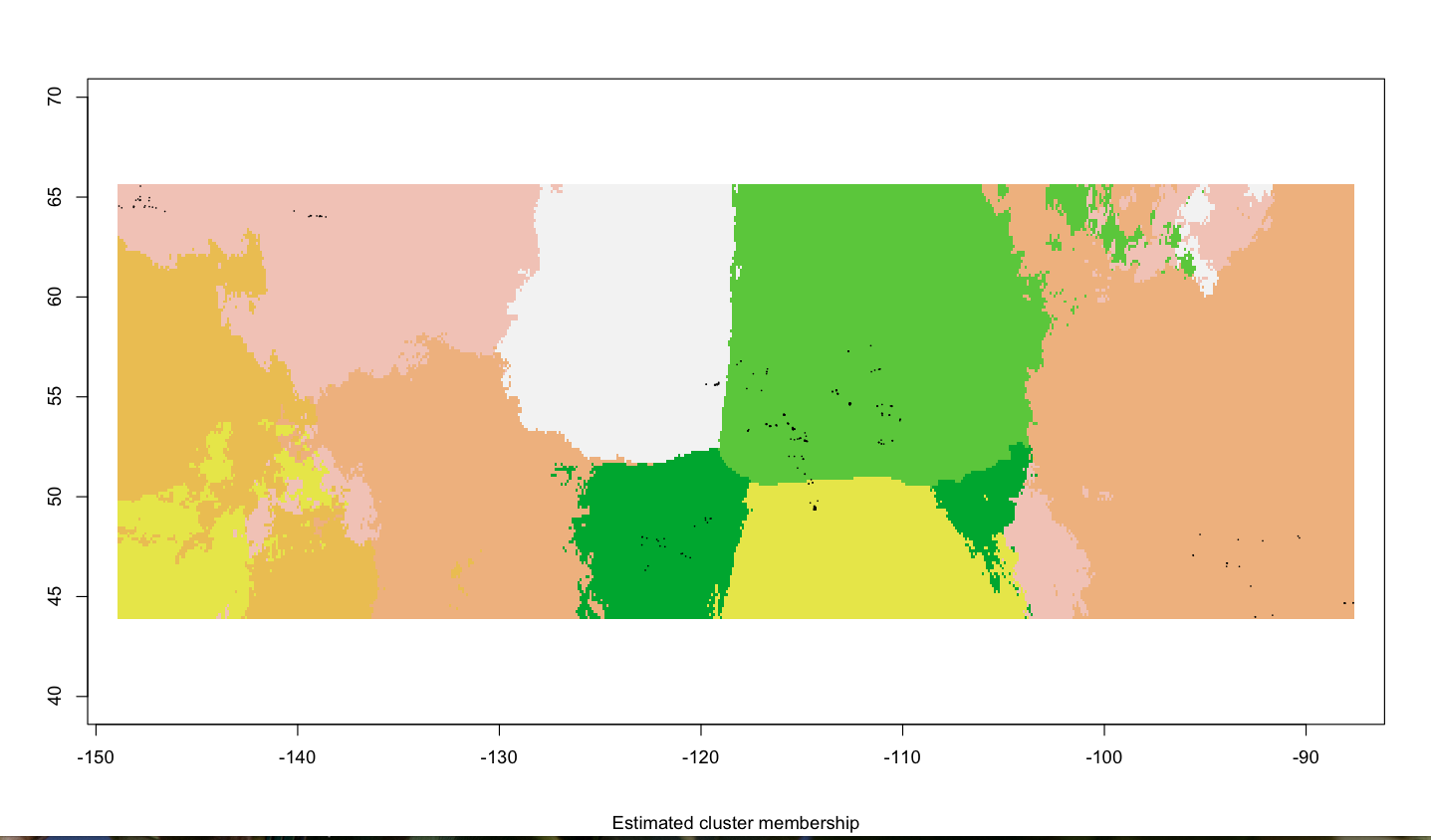
**

Longitude

Latitude

**b)**

AK


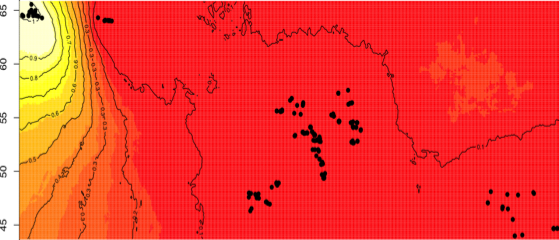

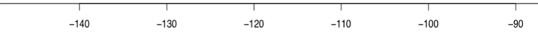

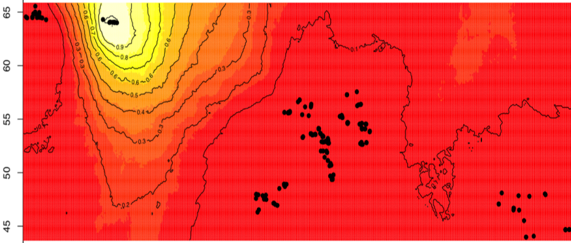

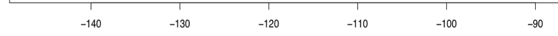

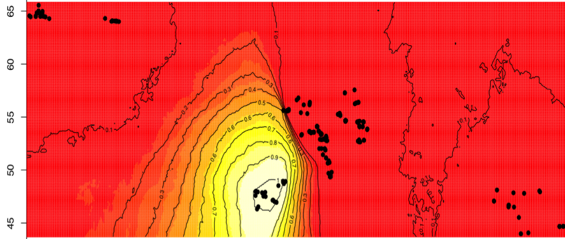

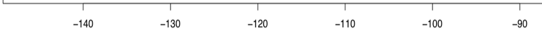

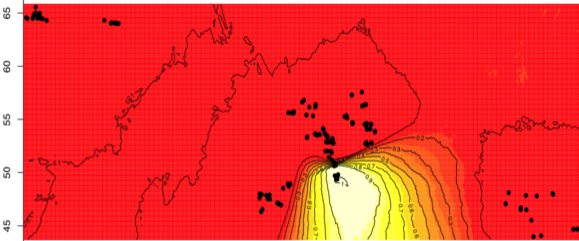

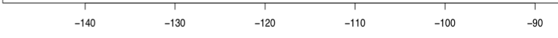


YT

WA

CP


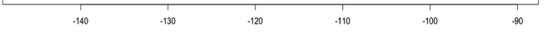

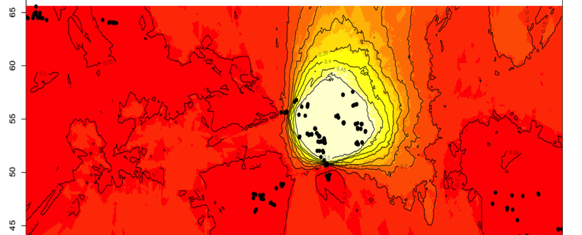

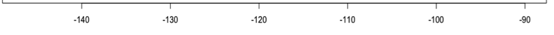

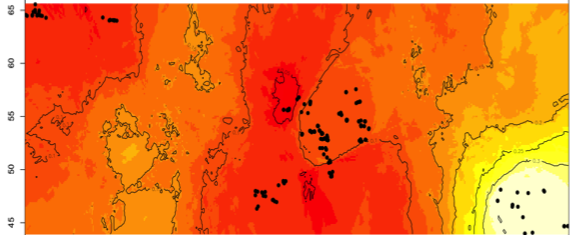

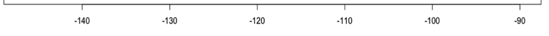

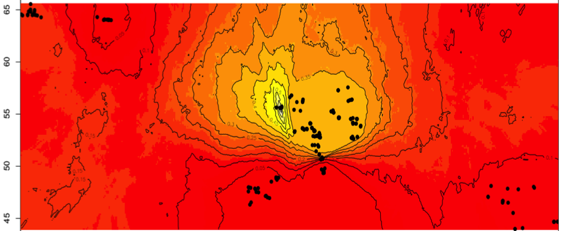


Alberta (main)

Great Lakes

GP

**Figure S4.** Ruffed Grouse population structure as inferred by GENELAND v4.0.6 (Guillot et al. 2005) from eight microsatellite loci. (a) The estimated cluster membership map shows the assignment of samples to K=7 clusters with clusters color-coded and samples from each population plotted onto the map in geographic space. (b) The posterior probability maps of cluster membership are shown here. The gradient lines (i.e. contour lines) connect points of the same degree of differentiation to show genetic clines, and the clusters are plotted in geographic space with latitude and longitude in decimal degrees on the y- and x-axes respectively.


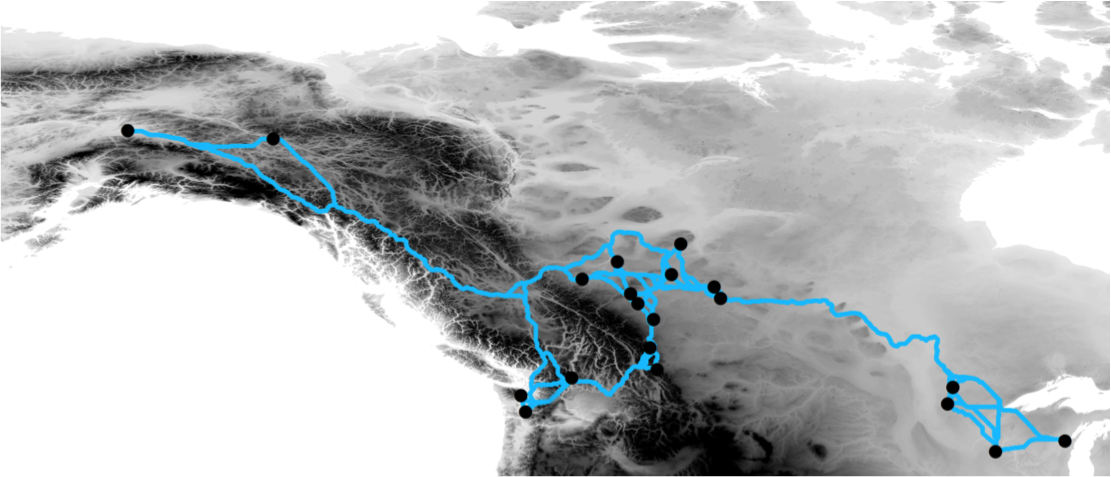

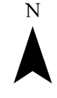

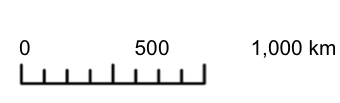

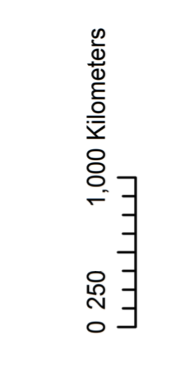


**Figure S5.** The least cost paths (LCP) between the 15 sampled populations of Ruffed Grouse showing the most likely dispersal routes between populations based on the preferred environmental conditions of the species as inferred by the SDM.
